# Evidence that immunization with TP0751, a bipartite *Treponema pallidum* lipoprotein with an intrinsically disordered region and lipocalin fold, fails to protect in the rabbit model of experimental syphilis

**DOI:** 10.1371/journal.ppat.1008871

**Published:** 2020-09-16

**Authors:** Amit Luthra, Jairo M. Montezuma-Rusca, Carson J. La Vake, Morgan LeDoyt, Kristina N. Delgado, Timothy C. Davenport, Mary Fiel-Gan, Melissa J. Caimano, Justin D. Radolf, Kelly L. Hawley

**Affiliations:** 1 Department of Medicine, UConn Health, Farmington, United States of America; 2 Division of Infectious Diseases, UConn Health, Farmington, United States of America; 3 Department of Pediatrics, UConn Health, Farmington, United States of America; 4 Department of Pathology, Hartford Hospital, Hartford, United States of America; 5 Department of Molecular Biology and Biophysics, UConn Health, Farmington, United States of America; 6 Department of Genetics and Genome Sciences, UConn Health, Farmington, United States of America; 7 Department of Immunology, UConn Health, Farmington, United States of America; 8 Division of Infectious Diseases and Immunology, Connecticut Children’s, Hartford, United States of America; University of Montana, UNITED STATES

## Abstract

Deconvolution of syphilis pathogenesis and selection of candidate syphilis vaccinogens requires detailed knowledge of the molecular architecture of the *Treponema pallidum* outer membrane (OM). The *T*. *pallidum* OM contains a low density of integral OM proteins, while the spirochete’s many lipoprotein immunogens are periplasmic. TP0751, a lipoprotein with a lipocalin fold, is reportedly a surface-exposed protease/adhesin and protective antigen. The rapid expansion of calycin/lipocalin structures in the RCSB PDB database prompted a comprehensive reassessment of TP0751. Small angle X-ray scattering analysis of full-length protein revealed a bipartite topology consisting of an N-terminal, intrinsically disordered region (IDR) and the previously characterized C-terminal lipocalin domain. A DALI server query using the lipocalin domain yielded 97 hits, 52 belonging to the calycin superfamily, including 15 bacterial lipocalins, but no Gram-negative surface proteins. Surprisingly, Tpp17 (TP0435) was identified as a structural ortholog of TP0751. *In silico* docking predicted that TP0751 can bind diverse ligands along the rim of its eight-stranded β-barrel; high affinity binding of one predicted ligand, heme, to the lipocalin domain was demonstrated. qRT-PCR and immunoblotting revealed very low expression of TP0751 compared to other *T*. *pallidum* lipoproteins. Immunoblot analysis of immune rabbit serum failed to detect TP0751 antibodies, while only one of five patients with secondary syphilis mounted a discernible TP0751-specific antibody response. In opsonophagocytosis assays, neither TP0751 nor Tpp17 antibodies promoted uptake of *T*. *pallidum* by rabbit peritoneal macrophages. Rabbits immunized with intact, full-length TP0751 showed no protection against local or disseminated infection following intradermal challenge with *T*. *pallidum*. Our data argue that, like other lipoprotein lipocalins in dual-membrane bacteria, TP0751 is periplasmic and binds small molecules, and we propose that its IDR facilitates ligand binding by and offloading from the lipocalin domain. The inability of TP0751 to elicit opsonic or protective antibodies is consistent with a subsurface location.

## Author summary

Development of a syphilis vaccine requires knowledge of the molecular architecture of the *Treponema pallidum* outer membrane (OM). The *T*. *pallidum* OM contains a paucity of integral OM proteins, while the spirochete’s lipoprotein immunogens are periplasmic. TP0751, a lipoprotein with a lipocalin fold, is reportedly a surface-exposed adhesin/protease and protective antigen. The recent increase in calycin/lipocalin structures prompted a comprehensive re-examination of TP0751. TP0751 contains two domains—an N-terminal intrinsically disordered region (IDR) and the previously characterized C-terminal lipocalin. Query of the structural databases identified related bacterial lipocalins, none known surface molecules. *In silico* docking studies predicted that TP0751 binds diverse ligands along the rim of its eight-stranded β-barrel and this was confirmed in heme binding assays. TP0751 is poorly expressed compared to other *T*. *pallidum* lipoproteins and barely elicits antibodies in syphilis immune rabbits or patients with secondary syphilis. TP0751 antibodies did not promote uptake of spirochetes by rabbit peritoneal macrophages, and immunization of rabbits with intact, full-length TP0751 did not prevent local or disseminated infection. Our data indicate that TP0751 resides within the periplasmic space of *T*. *pallidum* where it binds small molecules; we propose that the IDR facilitates ligand binding by and offloading from the lipocalin domain.

## Introduction

Syphilis is a multi-stage, sexually transmitted infection renowned for its protean clinical manifestations and protracted, often lifelong, course [1, 2]. The complex natural history of the disease reflects the invasiveness, immunoevasiveness, and inflammatory potential of its etiologic agent, the pathogenic spirochete *Treponema pallidum* subsp. *pallidum* (*T*. *pallidum*) [3, 4]. Although *T*. *pallidum* remains exquisitely susceptible to penicillin after more than seven decades of use [5], in the new millennium, syphilis has undergone a dramatic resurgence in the United States, particularly among men who have sex with men [6]. Syphilis also poses a serious and growing threat to global health; the World Health Organization estimates an annual worldwide incidence of approximately six million cases and 350,000 adverse outcomes in pregnancy due to mother-to-child transmission [7]. These alarming trends underscore the urgent need for a vaccine with global efficacy [8, 9].

*T*. *pallidum* is an extracellular, dual-membrane bacterium and obligate human pathogen [10, 11]. It is generally believed that clearance of spirochetes is mediated by antibodies that promote uptake and degradation by professional phagocytes, principally activated macrophages [12–15]. Strategies to elicit protective antibodies by immunization with recombinant treponemal proteins require detailed knowledge of the molecular architecture of the spirochete’s outer membrane (OM) as well as the membrane topology and structure of candidate vaccinogens [16]. To support its unique parasitic lifestyle [4], *T*. *pallidum* has evolved a cell envelope whose ultrastructure and composition differ markedly from those of prototypical diderms, such as *Escherichia coli* [16–18]. In addition to lacking lipopolysaccharide [19], the *T*. *pallidum* OM is a fluid and fragile lipid bilayer with a much lower density of membrane-spanning proteins than its Gram-negative counterparts [16, 18, 20]. *T*. *pallidum* also expresses an abundance of lipoproteins [19, 21, 22], many of which are highly immunogenic [3, 21, 23, 24]. An extensive body of evidence obtained using a variety of methodologies indicates that the syphilis spirochete’s lipoprotein immunogens reside within the periplasm [4, 16, 18]. Tpp47 (TP0574), the first *T*. *pallidum* protein shown to be lipid-modified [25], was biochemically and structurally defined as a penicillin-binding protein with DD-carboxypeptidase activity involved in peptidoglycan remodeling [26, 27]. X-ray crystallographic analyses revealed that many *T*. *pallidum* lipoproteins are substrate-binding proteins for ABC transporters that shuttle nutrients across the cytoplasmic membrane (CM) [4, 28–32]. For a lipoprotein to functionally interact with the CM permease of an ABC transporter, the protein moiety must be lipid-anchored to the periplasmic leaflet of the CM. A key implication of these findings is that the vast majority of antibodies generated during syphilitic infection are directed against subsurface proteins, in essence, acting as decoys to confound host defenses. From the vaccine standpoint, antibodies elicited against these lipoproteins will not be protective because they are not surface-exposed. The paucity of surface antigenic targets is the ultrastructural basis for *T*. *pallidum*’s impressive capacity to evade innate and adaptive responses, hence, its designation as “the stealth pathogen” [3, 4].

Nevertheless, the above studies do not preclude the possibility that *T*. *pallidum* expresses some lipoproteins on its surface. TP0136, a fibronectin-binding protein with sequence variability among *T*. *pallidum* strains, was reported to be surface-exposed in the Nichols strain by immunoelectron microscopy [33, 34]. Chan *et al*. [35] found that Tpp17 (TP0435) enhanced cytadhesiveness in “gain of function” experiments when expressed in avirulent *B*. *burgdorferi*, and they also observed modest surface labeling of *T*. *pallidum* by immunoelectronmicroscopy. Because of the fragile nature of the *T*. *pallidum* OM, surface localization experiments are technically challenging and must carefully control for OM integrity as well as antibody specificity [16]. In our hands, using our sensitive gel microdroplet system for immunofluorescence analysis [36, 37], neither TP0136 nor Tpp17 localized to the surface of intact treponemes, while both were readily detected in organisms whose OMs were disrupted by mild detergent treatment [37, 38]. Furthermore, Tpp17 is a structural ortholog for the N-terminal domain of *E*. *coli* NlpE [39], a periplasmic lipoprotein involved in sensing OM stress [40]. First identified as a laminin-binding adhesin [41], TP0751 also has been reported to be a zinc-dependent metalloprotease (hence, its designation “pallilysin”) that forms a biologically active complex with TP0750, which contains a Von Willebrand factor type A domain [42–44]. TP0751 heterologously expressed in *B*. *burgdorf*eri could be detected by flow cytometry on the surface of Lyme disease spirochetes and promoted their attachment to HUVEC monolayers [45, 46]. Importantly, TP0751 has emerged as a candidate syphilis vaccinogen. Immunization of rabbits with TP0751 attenuated lesion development and reduced spirochete dissemination following intradermal challenge with *T*. *pallidum* [47].

In the absence of methods for genetically manipulating *T*. *pallidum*, structural biology has become an essential tool for inferring cellular location and function of treponemal proteins [4]. The solved X-ray structure of residues 97–227 of TP0751 revealed a lipocalin fold [45]. Found in all kingdoms of life, lipocalins (a division of the calycin superfamily) are a family of proteins with great sequence diversity but conserved structure consisting of an eight-stranded, antiparallel β-barrel that transports, stores or sequesters small, usually lipophilic, molecules [48, 49]. In Gram-negative bacteria, lipocalins are soluble or lipid-modified periplasmic proteins [50–54]. The recent rapid expansion of lipocalin structures, many liganded, in the RCSB PDB database [49, 54, 55] prompted us to undertake a comprehensive reassessment of TP0751. Rather than being a surface adhesin/protease and target of protective antibodies, our collective data indicate that TP0751 is an extremely low abundance periplasmic lipoprotein predicted to bind small molecules in characteristic lipocalin fashion. Furthermore, we propose that the protein’s previously unrecognized N-terminal, intrinsically disordered region (IDR) promotes binding and offloading of ligands from the C-terminal lipocalin domain. Unexpectedly, we found that the much more abundant Tpp17 has a lipocalin fold resembling that of TP0751, suggesting functional similarity between these two lipoproteins.

## Results

### SAXS structure of TP0751 reveals a dynamic, two-domain architecture

The nonacylated, full-length TP0751 constructs produced by Cameron and co-workers [42, 45] were highly susceptible to proteolytic degradation. They concluded that TP0751 has intrinsic proteolytic activity and is subject to autolysis [42]. To circumvent this problem for their structural studies, they produced a truncated version of the protein (residues 78–237) for X-ray crystallography and solved the structure for residues 97–228 (TP0751^97-228^, PDB ID: 5JK2-F) [45]. In our hands, however, a full-length C-terminal His_6_-tagged construct (TP0751^25-237^) was stable and migrated by SDS-PAGE with an apparent molecular weight of ~31 kDa (**S1A Fig**); as previously suggested [42], the large number (18) of proline residues throughout the protein likely explains the slower than expected (24.6 kDa) electrophoretic migration. By size-exclusion chromatography, however, TP0751^25-237^ purified to homogeneity eluted as a monomer with a molecular weight of 25 kDa (**S1B Fig**).

**Fig 1:**
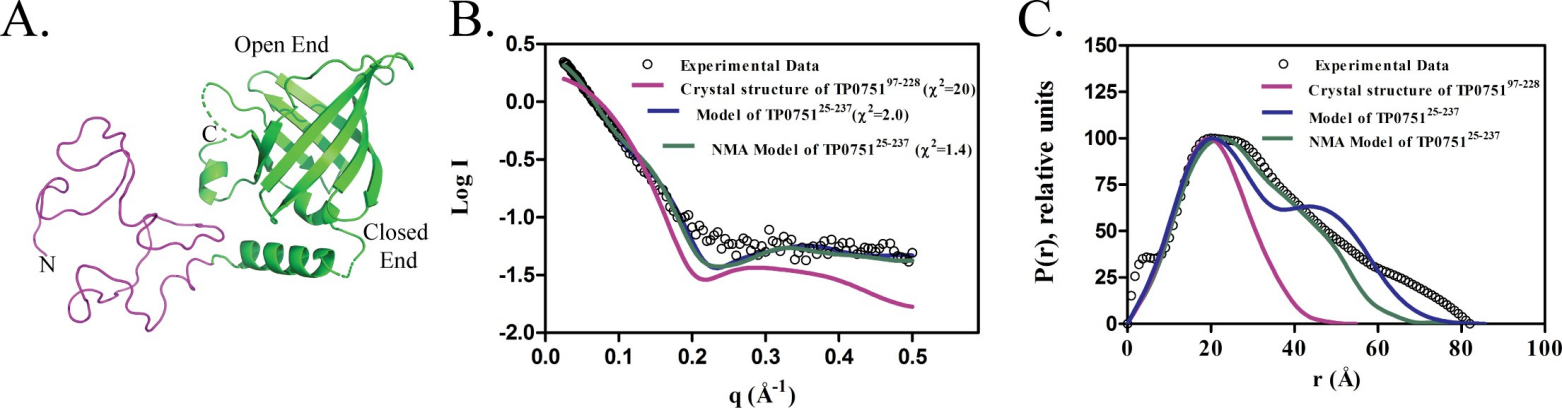
SAXS analysis of full-length TP0751. **(A)** Ribbon diagram of the bipartite structural model of full-length TP0751 generated using the intensive mode of Phyre2 [56]. The unstructured N-terminal region (residues 25–96) and C-terminal lipocalin domain (residues 97–237) are shown in pink and green, respectively. **(B)** Plots showing the log of the scattering intensity (I) as a function of momentum transfer (q = 4πsin(θ)/λ). The black circles are SAXS experimental data; the colored curves are theoretical scattering profiles calculated from different structural models using FoXS [123]. The χ2 shown in parentheses indicate the fit of the theoretical scatterings to the experimental SAXS data. **(C)** Comparison of the normalized inter-atomic pairwise distribution function (P(r)), computed from the experimental SAXS data (black circles) and different 3D models (colored lines). The P(r) functions show that TP0751^25-237^ has a bipartite architecture with a diameter of approximately 75Å.

**Fig 2:**
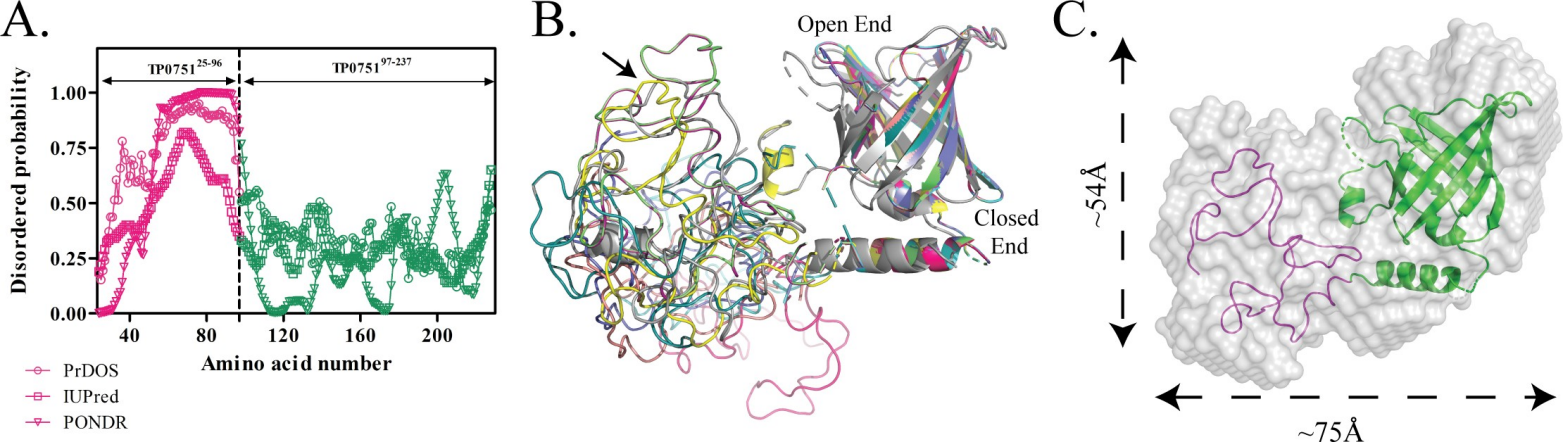
The N-terminal region of TP0751 is intrinsically disordered. **(A)** Prediction of disorder propensity along the length of TP0751^25-237^ using IUPred2 [58], PrDOS [57], PONDR [131]. The dashed line represents the domain boundary of TP0751^25-237^. (**B)** Ribbon diagrams show ten NMA- generated conformations of the bipartite model of TP0751^25-237^. The best optimized is shown in yellow and indicated by the arrow. **(C)** *Ab initio* reconstruction of the molecular envelope (gray surface) of TP0751^25-237^ calculated from the SAXS data and overlaid on the NMA optimized structural model.

We next performed small angle x-ray scattering (SAXS) experiments to obtain the molecular envelope of full-length TP0751. To facilitate interpretation of the SAXS data, we generated a three-dimensional (3D) structural model of TP0751^25-237^ using the intensive mode of Phyre2 [56] (**Fig 1A**). This model predicts that TP0751 is bipartite and consists of a disordered N-terminal region (TP0751^25-96^) and a structured C-terminal domain (TP0751^97-237^, PDB:5JK2). We then calculated theoretical scattering curves and intra-particle distance distribution functions (P(r)) from the PDB files of the crystal structure and the 3D structural model of TP0751^25-237^. The experimental SAXS data showed good agreement (χ^2^ = 2.0) with the theoretical scattering curve derived from our full-length structural model but not with the crystal structure alone (χ^2^ = 20) (**Fig 1B and 1C**). Analysis of TP0751^25-237^ using PrDOS [57], IUPred [58] and PONDR [59] predicts that TP0751^25-96^ is intrinsically disordered (**Fig 2A**). To confirm this prediction, we used normal mode analysis (NMA) [60] to examine the conformational entropy of TP0751^25-237^. NMA revealed that structural plasticity is restricted to the N-terminal region (**Fig 2B**); one NMA-optimized structural model of TP0751^25-237^ was in best agreement with the experimental scattering curve (*χ*^2^ = 1.4) and P(r) distribution (**Fig 1B** and **1C**, respectively). Finally, we generated an *ab initio* molecular envelope of TP0751^25-237^ by averaging ten independent Dammin beads models [61, 62] without enforcing any symmetry. The NMA-optimized structural model fits well into the SAXS envelope (**Fig 2C**).

### TP0751 is a calycin with eukaryotic as well as prokaryotic structural orthologs

Database searches failed to identify sequences related to the disordered N-terminal region of TP0751; we, therefore, focused our attention on its C-terminal lipocalin fold. Upon examination of the TP0751^97-228^ structure, we found a Gly-X-Trp/Arg signature motif for calycins, a superfamily of 8 and 10 anti-parallel, stranded hydrophilic β-barrels that includes lipocalins [63]. Trp-Arg aromatic-amine interactions within Gly-X-Trp/Arg motif stabilize the calycin β-barrel [64]; in TP0751, this motif consists of Arg226, located on β-strand 8, and Trp127 on β-strand 1 (**S2 Fig**). Historically, lipocalins are classified as either ‘kernel’ or ‘outlier’ depending on the presence or absence, respectively, of three ‘short conserved regions’ or SCRs [65, 66]. According to this scheme, TP0751 would be considered an outlier because it lacks SCR-2.

Our initial search for bacterial lipocalin orthologs of TP0751^97-228^ failed to identify any sequence orthologs in multiple databases when *Treponema* species were excluded. This was not surprising given the low degree of sequence similarity (~10%) known to exist within the calycin/lipocalin superfamily [67, 68]. Structural relationships often can be discerned among proteins with limited sequence relatedness [69, 70]. Therefore, we next searched for structural orthologs to glean new insights into the function and location of TP0751 in *T*. *pallidum*. A query of the RCSB Protein Data Bank (https://www.rcsb.org/) via the DALI server [71] using the TP0751^97-228^ structure (PDB ID: 5JK2-F) yielded 97 hits with Z-scores ranging from 10.0 (top hit) to 2.4 (lower limit of significance) (**S1 Table**). By manual curation, we determined that 52 of the hits are members of the calycin superfamily; **Table 1** contains a summary of their Z-scores, RMSD values, and, when known, inbound crystallized ligands. Interestingly, the overwhelming majority (37 of 52) of the calycin orthologs are eukaryotic and include fatty acid-binding proteins (FABPs), retinoic acid-binding proteins, and allergens. Fifteen of the 52 are bacterial lipocalins; 10 of these are uncharacterized (**Table 1**). Of the remaining five, three are periplasmic proteins in Gram-negatives: *Vibrio parahaemolyticus* VtrA (PDB ID: 5KEW, liganded with the bile salt taurodeoxycholate) [54], *E*. *coli* Blc (PDB ID: 2ACO, liganded with the *trans-*fatty acid vaccenic acid) [53], and the N-terminal domain of *E*. *coli* NlpE (PDB ID: 2Z4H, also referred to CutF) [72]. A fourth is YxeF (PDB ID: 2JOZ) from the monoderm *Bacillus subtilis* [73]. The fifth, unexpectedly, is the *T*. *pallidum* lipoprotein Tpp17 (TP0435, PDB ID: 4U3Q) [39], already designated by UniProt to contain an NlpE domain (Pfam PF04170) [39, 72]. Under normal growth conditions, NlpE in *E*. *coli* is anchored to the inner leaflet of the OM; during cell envelope stress, it accumulates on the periplasmic side of the CM, where it activates the CpxAR two-component system [40]. Notably absent from our DALI search results was Nitrophorin 4, the ‘outlier’ lipocalin used by Parker *et al*. [45] as a comparator to TP0751^97-228^.

**Table 1:** Calycin/lipocalin structural orthologs of TP0751.

Protein	PDB: ID^1^	Z-score	RMSD (Å)^2^	Number of antiparallel β strands	Interacting Ligand^3^
Sahs1	5xn9	9.1	3	10	
Sahs4	5z4g	8.9	3.3	10	
Fatty acid binding protein, Epidermal	5ur9	8.9	3.3	10	2,4-diphenylcyclobutane-1-carboxylic acid
Uncharacterized protein Bt_0846 ^¥^	**2m4l**	**8.8**	**2.2**	**8**	
Uncharacterized protein ^¥^	**4q51**	**8.4**	**3**	**8**	
Retinoic acid binding protein	1epa	8.3	2.8	8	
17 kDa lipoprotein (Tpp17) ^¥^*	**4u3q**	**7.9**	**3.1**	**8**	
Hypothetical protein ^¥^	**4iab**	**7.7**	**3.1**	**8**	**Di(Hydroxyethyl)Ether**
Uncharacterized protein ^¥^	**2mhd**	**7.6**	**3**	**8**	
Bla G 4 allergen	4n7c	7.6	2.9	8	Citric Acid
Antenna protein	5hi8	7.4	3.2	10	
Complement component 8	2qos	7.3	3.2	8	
Thap domain containing protein 4	3ia8	7.3	3.6	10	Heme
Retinol binding protein	2rcq	7.3	2.6	10	Retinol
Biogenic amine binding protein	4ge1	7.2	2.8	8	Ethanamine
Per A 4 allergen	3ebw	7.1	2.6	8	Derivative of Ethanol
Lipocalin Ai4	5ha0	7.1	3.4	8	Derivative of Tetraenoic Acid
Gastrotropin	5l8i	6.9	3.4	8	Cholic Acid
Lipocalin allergen	4odd	6.7	2.9	8	
Hypothetical protein	2o62	6.7	3.6	10	
Yxef ^‡^*	**2joz**	**6.6**	**3.4**	**8**	
Vtra protein ^¥^*	**5kew**	**6.6**	**2.6**	**8**	**Bile Salt Taurodeoxycholate**
Cellular retinoic acid binding protein	6nnx	6.6	3.3	10	Retinol
Putative uncharacterized protein ^¥^	**5byp**	**6.5**	**2.8**	**8**	
Outer membrane lipoprotein Blc ^¥^*	**2aco**	**6.5**	**2.8**	**8**	**Vaccenic Acid**
Hypothetical Protein Bt_0869 ^¥^	**3hty**	**6.5**	**2.8**	**8**	
p-coumaric acid decarboxylase	2gc9	6.4	3.2	9	Citric Acid
Transthyretin	1qab	6.4	3.0	8	Retinol
Bile acid binding protein	3elz	6.4	2.8	10	Cholic Acid
Fatty acid binding protein homolog	6i9f	6.4	3.5	10	Oleic Acid
Extracellular fatty acid binding protein	3sao	6.3	2.6	8	Propyl Tetradecanoate
Hypothetical Protein ^‡^	**4kh8**	**6.3**	**3.6**	**8**	
Odorant binding protein 2A	4run	6.3	3.0	8	Citrate Anion
Crustacyanin	1i4u	6.2	2.7	8	2-Methyl-2,4-pentanediol
Phycoerythrin lyase	4tq2	6.2	3.4	10	Hexane-1,6-Diol
Bovine β lactoglobulin A	1bso	6	3.2	8	12- Bromododecanoic Acid
Lipoprotein	3lhn	6	3.0	8	
Hp1028 ^¥^	**4inn**	**6**	**3.0**	**8**	**Hexaoxaicosane-1,2-diol**
Alpha1 acid glycoprotein 2	3apx	5.9	3.2	8	Dimethylpropan-1-amine
Lipoprotein^‡^	**3ge2**	**5.7**	**2.9**	**8**	
Allergen can F 2	3l4r	5.7	3.4	8	
Uncharacterized protein^¥^	**4zgf**	**5.7**	**3.1**	**8**	
Violaxanthin deepoxidase	3cqr	5.6	3.1	8	
Apolipoprotein M	2wew	5.6	3.3	8	Myristic Acid
Histamine binding protein	1qft	5.4	3.6	8	Histamine
Insecticyanin	1z24	5.3	2.7	8	Biliverdin
Fatty acid binding protein	2mo5	5.2	4.1	10	Oleic Acid
Lipocalin	3brn	5.2	3.9	8	Serotonin
Phycocyanobilin lyase	4o4s	5	3.7	10	Phycocynobilin
N-terminal domain of NlpE ^¥^*	**2z4h**	**4.9**	**2.4**	**7**	
Darcin	2l9c	4.1	4.7	8	
Fatty acid binding protein	2n93	3.9	3.5	10	

^1^ RCSB Protein database entry code.

^2^ Root-mean-square deviation (RMSD) of C-α atoms in the least-squares superimposition of the structurally equivalent C-α atoms.

^¥^ Gram-negative proteins.

^**‡**^ Gram-positive proteins

* Functionally characterized bacterial proteins

### *In silico* docking predicts that TP0751 binds small molecules along the barrel rim

**Fig 3:**
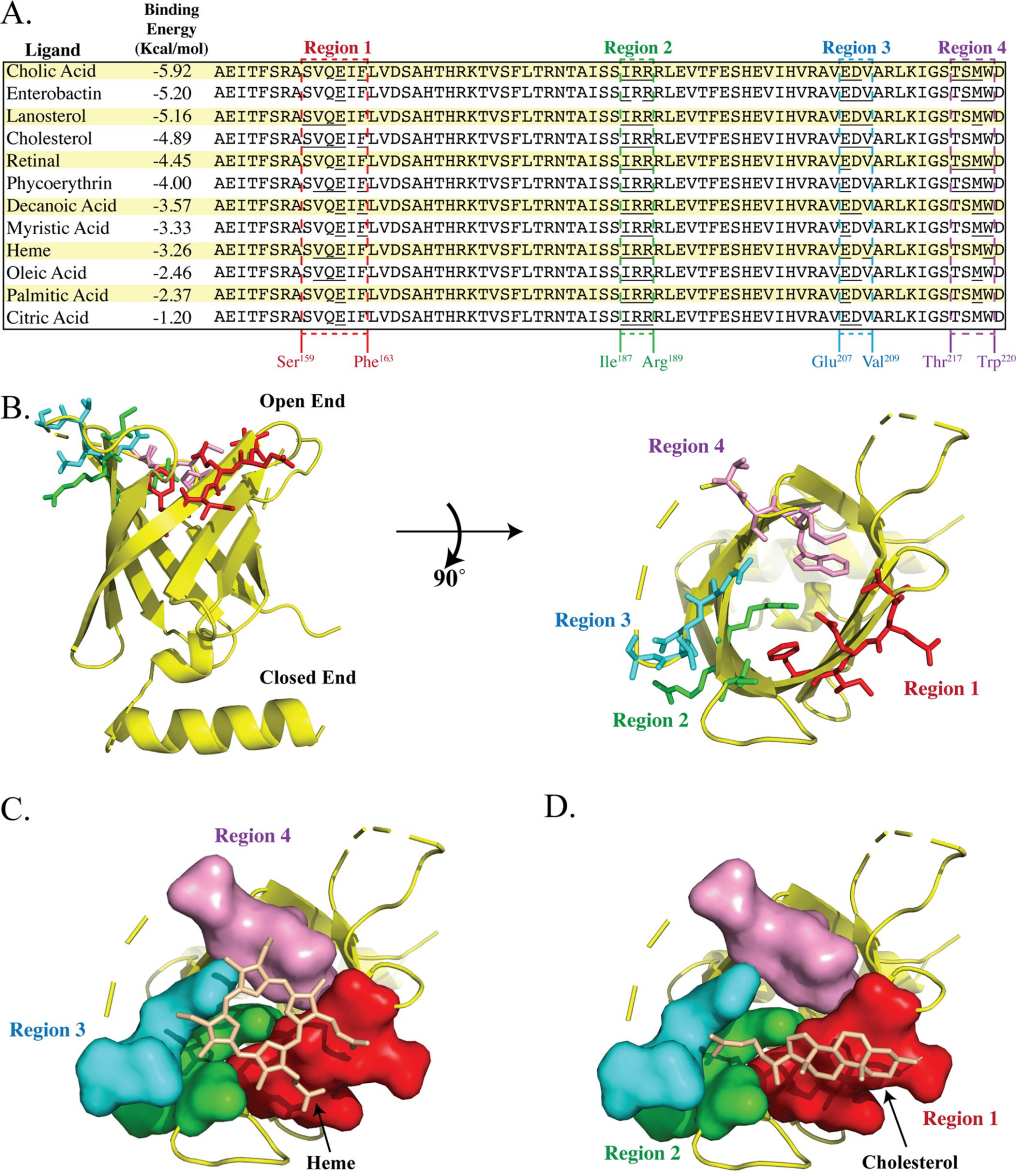
Structural mapping of the predicted ligand-binding regions of TP0751^97-228^. **(A)** The left panel shows selected ligands and their docking energies. Dashed boxes indicate the predicted binding regions; residues predicted to interact with individual ligands are underlined. (**B)** Close-up of the predicted ligand-binding sites. The residues predicted to form the four binding regions are depicted as sticks and colored as in panel A. Molecular docking snapshots of predictions for binding of **(C)** heme and **(D)** cholesterol.

TP0751 has been studied extensively for its laminin-binding properties [41, 42]. In peptide mapping experiments, Parker *et al*. [45] identified four regions, distributed around the lipocalin barrel, that mediate TP0751-laminin interactions. Of these, 'peptide 10' (p10) recently also was shown to interact with the laminin receptor (LamR) [74]. Mapping of p10 onto a 3D model for TP0751^97-228^ reveals that it corresponds to two positively charged β strands (strands 5 and 6) with adjoining loop (**S3 Fig**). Since calycins/lipocalins typically bind small molecules (**Table 1**), often hydrophobic in nature, on or within the barrel [49, 75, 76], binding sites on the external façade of the barrel are atypical. Consequently, we used a bioinformatics approach to explore biologically plausible alternative binding scenarios. COACH [77, 78] and 3DLigandSite [79], programs that search curated databases for biologically relevant ligands [78], predicted that TP0751 can bind retinol and heme, respectively. However, scrutiny of the outputs of these two programs revealed that both created steric clashes with residue side chains that project into the barrel cavity (**S3A and S3B Fig**). These results prompted us to examine the interior of the TP0751^97-228^ barrel; CASTp [80] determined that the cavity has a relatively small volume (34.9 Å^3^). We next performed rigid-body docking experiments using AutoDock [81] to interrogate the entire barrel for potential binding sites. The grid used for these *in silico* analyses encompasses the three laminin binding peptides (p6, p10, p11) previously mapped to the TP0751^97-228^ barrel [45] (**S3C** and **S3D Fig**). The compounds evaluated included known calycin ligands (**Table 1**) as well as molecules of physiological relevance for *T*. *pallidum* (*e*.*g*., fatty acids, cholesterol, and heme), an extreme auxotroph [19, 82, 83]. Interestingly, the selected ligands were predicted to bind with comparable free energies (**Fig 3A**), suggesting that TP0751 may bind multiple ligands. To identify interacting amino acids for each ligand, we used Ligplot+ [84] to examine the docked PDB files, which then were aligned (**Fig 3A**) and overlaid on the TP0751^97-228^ structure. As shown in **Fig 3B**, the interacting residues are distributed in four regions along the rim of the barrel. **Fig 3C** and **3D** depict the predicted docked structures for heme and cholesterol.

### The lipocalin domain of TP0751 binds heme

**Fig 4:**
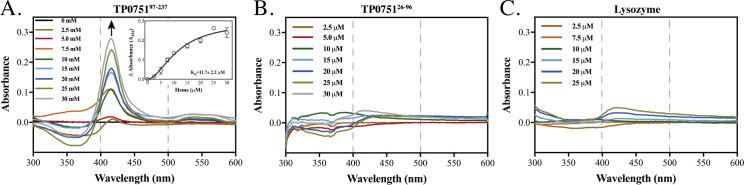
The lipocalin domain of TP0751 binds heme. UV/Vis differential spectra of (A) TP0751^97-237^ (5 μM), (B) TP0751^25-96^ (5 μM), and (C) lysozyme (10 μM) incubated with graded concentrations of heme. The arrow in panel A denotes absorption maxima; the dashed lines at 400 and 500 nm in all three panels denote the spectral range for band broadening [132]. The changes in absorbance of the Soret peak (416 nm) plotted against heme concentrations (0–30 μM) were used to determine the dissociation constant (K_d,_ 11.7 ± 2.2 μM) for TP0751^97-237^, using a one-site binding model with Hill slope (Inset, panel A).

As an initial step towards substantiating the *in silico* docking calculations, we investigated the ligand-binding ability of TP0751’s lipocalin domain. We chose to study heme because of its aqueous solubility and because it is well recognized as a potential source of iron for bacterial pathogens [4]. We spectrophotometrically assessed the affinities of both domains of TP0751 (5μM each), along with lysozyme (10 μM) as a negative control [85], in heme titration experiments (0–30 μM) at pH7.0 over a spectral range from 300 nm to 700 nm. TP0751^97-237^ displayed a K_d_ value of 11.7 ± 2.2 μM and a Soret-band shift at 416 nm (**Fig 4A**), while TP0751^25-96^ and lysozyme did not exhibit any binding (**Fig 4B** and **4C**, respectively). The solitary Soret peak at ~416 nm for TP0751^97-237^ indicates a single heme-binding site.

### The lipocalin domain of TP0751 and Tpp17 have similar structural topologies

**Fig 5:**
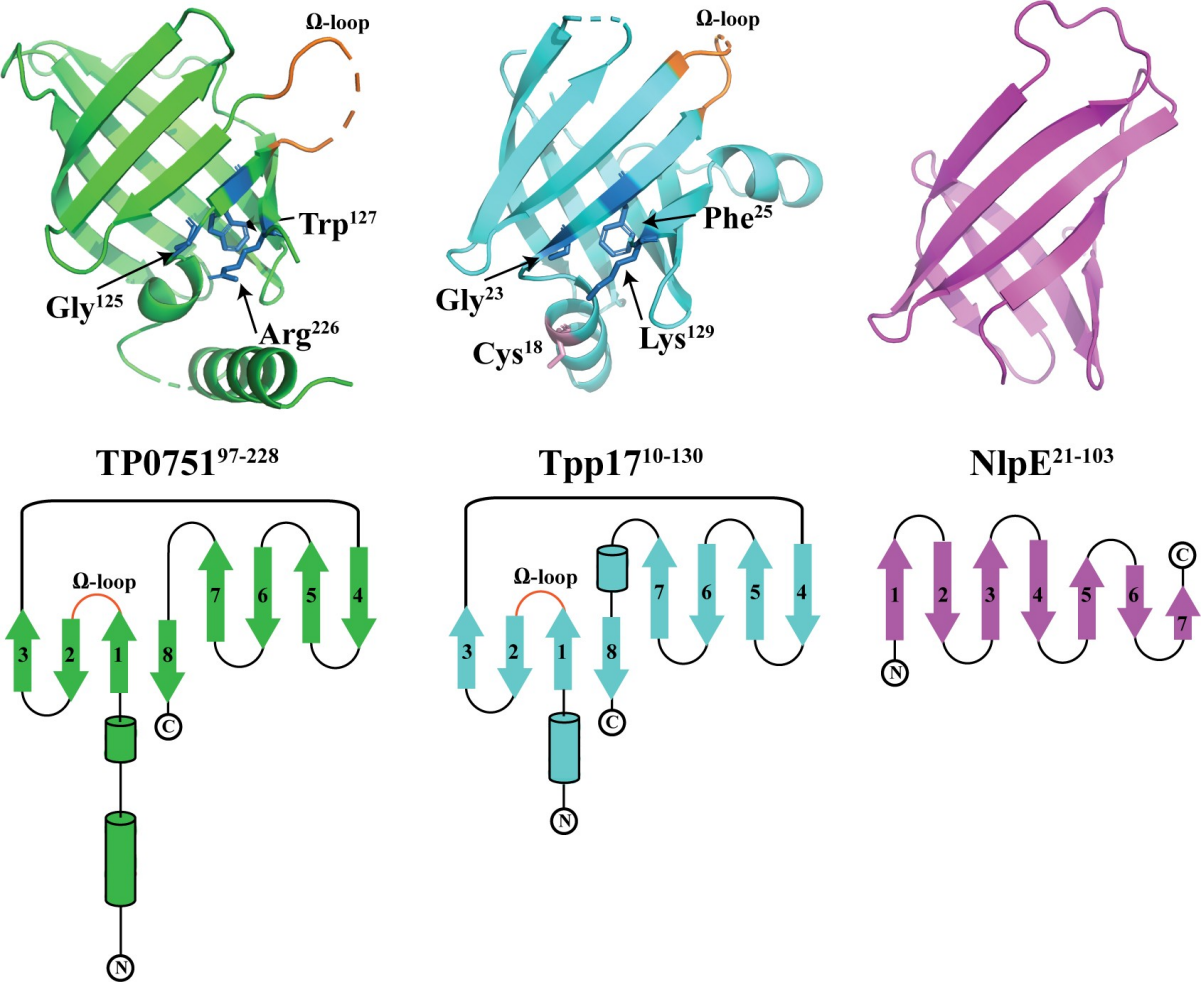
TP0751^97-228^ and Tpp17 share similar structural topologies. The upper panel shows ribbon diagrams for the structures of TP0751^97-228^ (PDB ID: 5JK2), Tpp17 (PDB ID:4U3Q), and the N-terminal domain (residues 21–103) of *E*. *coli* NlpE (PDB ID:4Z4H) (all in the same orientation). The Ω-type loops are shown in orange. Residues of the calycin signature motif are shown as blue sticks and labeled. The lower panel presents secondary structure topology diagrams. β-strands and α-helices are represented by arrows and cylinders, respectively. N- and C-termini are indicated, and the ‘Ω-type’ loops shared by TP0751^97-228^ and Tpp17 are labeled.

Our DALI search brought to light a previously unrecognized structural relationship between the lipocalin domains of TP0751 and Tpp17, which share only 11.5% amino acid identity. Comparison of the crystal structures for TP0751^97-228^ and Tpp17 revealed that both consist of eight antiparallel β-strands with a Ω-type loop between strands 1 and 2 **(Fig 5** and **S4 Fig)**, a characteristic feature of lipocalins [73]. Like TP0751, Tpp17 contains a calycin structural signature motif and a small cavity (28 Å^2^) (**Fig 5, ribbon diagram**). Tpp17 is annotated as a member of the NlpE family based on a comparison with the N-terminal β-barrel domain (residues 21–103) of *E*. *coli* NlpE [39]. The latter, however, contains only 7 antiparallel β-strands and lacks both a calycin motif and a Ω-type loop (**Fig 5**). Moreover, compared to the N-terminal domain of NlpE, TP0751^97-228^ and Tpp17 have more similar electrostatic charges externally **(S5 Fig, front view**). Two differences between TP0751^97-228^ and Tpp17 are noteworthy. First, the rim of the TP0751 barrel is more positively charged (pI = 8.8) compared to the Tpp17 rim (pI = 5.7) (**S5 Fig, top view**). The second is the presence of two free cysteines (Cys18 and Cys34) in Tpp17 (**Fig 5, ribbon diagrams;** note that the electron density of Cys34 was not resolved in the Tpp17 structure), which enables formation of dimer and multimers *in vitro* and *in vivo* [39].

We subsequently compared at the sequence level TP0751^97-228^, NlpE (N-terminal domain), and Tpp17 with other bacterial lipocalins identified by the DALI search. Surprisingly, in the sequence-based phylogeny (**S6 Fig**), Tpp17 (Z score = 7.9) and TP0751 are located on distant branches, while the structurally less similar N-terminal domain of NlpE (Z score = 4.9) grouped with TP0751. This analysis reinforces the value of structure-based comparisons for inferring relationships among calycin/lipocalin superfamily members [67].

### Native TP0751 is an extremely low abundance lipoprotein in *T*. *pallidum*

**Fig 6:**
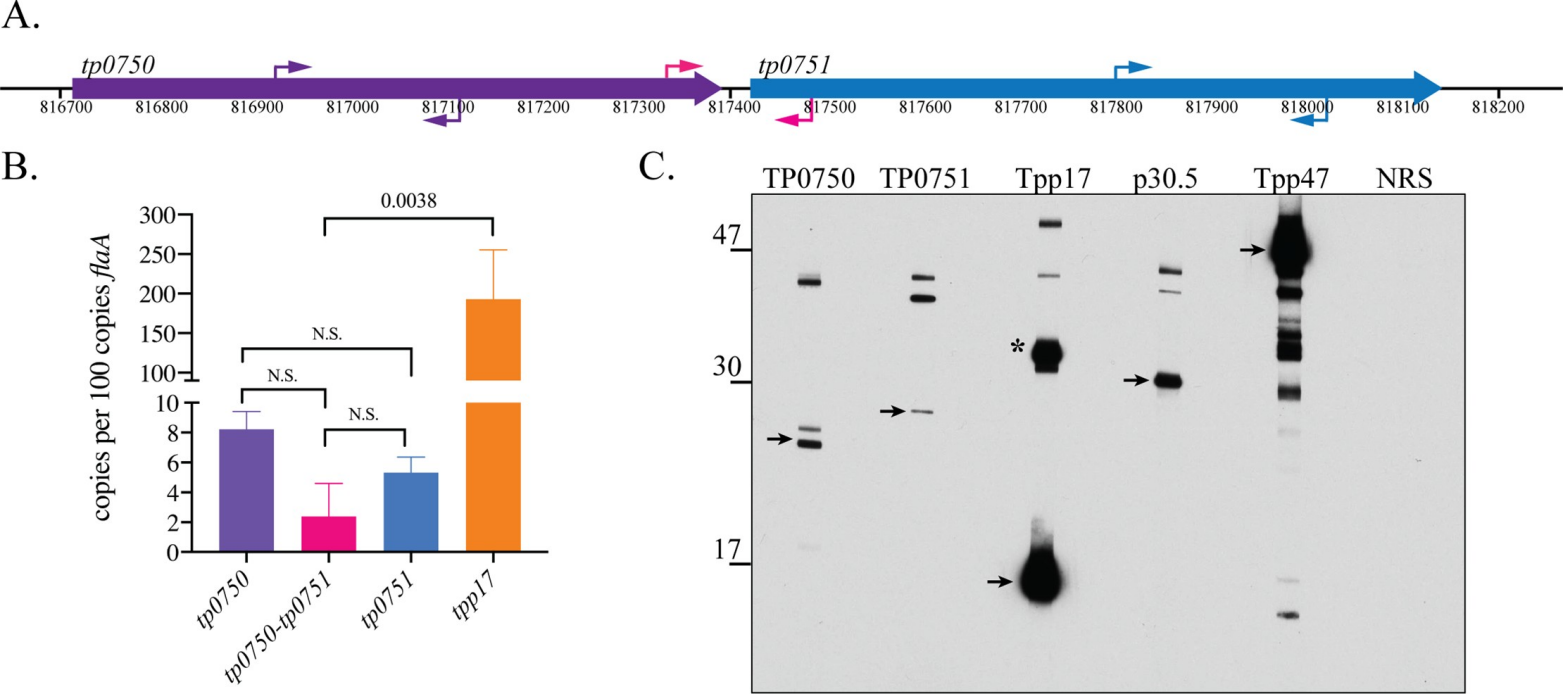
Native TP0751 is a low abundance lipoprotein in *T*. *pallidum*. (**A)** Schematic depiction of the *tp0750*-*tp0751* operon. Primers used for PCR amplification of *tp0750*, *tp0751* and the intergenic region (S2 Table) are indicated by purple, blue and pink arrows, respectively. **(B)** Transcript copy numbers normalized to *flaA*. Error bars indicate standard errors of the mean (3 biological replicates, assayed in quadruplicate). *p* values for pairwise comparisons were determined using a two-tailed *t* test. **(C)** Trinity Biotech MARBLOT *T*. *pallidum* lysate strips were incubated overnight at 4° C with polyclonal rat antisera (1:1,000) against TP0750, TP0751^25-237^, Tpp17, p30.5 (TP0453), and Tpp47 or normal rat serum (NRS) followed by goat anti-rat IgG HRP conjugate (1:30,000) for 1 h at RT. Strips were aligned and developed on a single sheet of film using the SuperSignal West Pico chemiluminescent substrate. Arrows indicate the presumptive monomers of TP0750 and TP0751, the known monomer of Tpp17, p30.5, and Tpp47. The asterisk indicates Tpp17 dimers [140]. The degradation products of Tpp47 have been described previously [87, 141].

Native TP0751 was not detected in *T*. *pallidum* in prior studies [42, 86], including by mass spectrometry, suggesting it is expressed at low abundance. To confirm this, we undertook an independent analysis of *tp0751*/TP0751 expression levels. *tp0751* is co-transcribed with *tp0750* in a bicistronic operon [44]. By qRT-PCR, we measured less than 10 copies of *tp0750*, *tp0751* or the intergenic region per 100 copies of *flaA* in *T*. *pallidum* Nichols freshly isolated from rabbit testes; transcript levels for *tp0751* were approximately 30-fold lower than for *tpp17* (**Fig 6A** and **6B**). We next assessed expression of TP0751 in *T*. *pallidum* by immunoblot using MARBLOT (Trinity Biotek) *T*. *pallidum* strips, which, because they are standardized, yield highly reproducible results. With dilutions of antisera normally used for detection of proteins that are well expressed in *T*. *pallidum*, we did not observe bands corresponding to monomeric TP0751 or TP0750. However, using a dilution (1:1,000) that resulted in obvious over-detection of both Tpp17 and another abundant periplasmic lipoprotein, Tpp47 [27, 87], we saw bands at ~25 kDa (TP0750) and ~27 kDa (TP0751), with the presumptive TP0751 band considerably less intense than that for TP0750 (**Fig 6C**). Although the TP0750 and TP0751 polypeptides have very similar calculated MWs (~23 kDa), the greater apparent MW of the presumptive native TP0751 monomer could be due to a combination of the anomalous migration of the protein (See **S1A Fig** and above) plus lipid modification (which would add ~1.5 kDa). The intensities of the presumptive TP0751 and TP0750 monomers were appreciably less than p30.5 (TP0453), a low-abundance lipoprotein anchored to the inner leaflet of the *T*. *pallidum* OM [88, 89]. With several antisera, we observed bands of uncertain identity not seen with normal rabbit serum.

### TP0751 induces a weak antibody response during rabbit and human syphilitic infection

**Fig 7:**
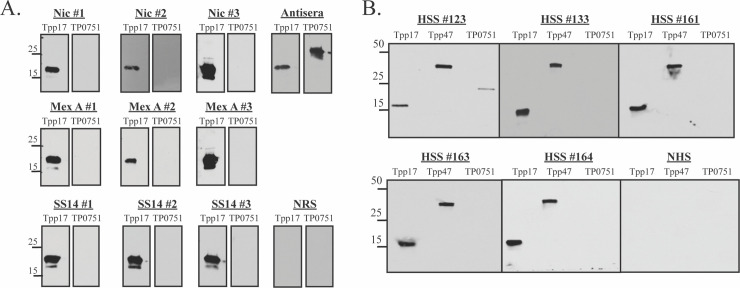
TP0751 weakly induces antibodies during experimental and human syphilis. Immunoblot reactivities of sera from **(A)** rabbits immune to re-infection with the Nichols, Mexico A or SS14 strains and **(B)** five HIV-negative patients with secondary syphilis (SS) against Tpp17, Tpp47 and TP0751^25-237^ (100 ng of each protein, 1° Ab 1:250). Molecular mass standards (in kilodaltons) are indicated on the left.

For TP0751 to contribute to natural immunity, it must elicit antibodies during syphilitic infection; antibody responses to TP0751 during experimental or human syphilis have not been described. We, therefore, performed immunoblotting experiments using sera from rabbits immune to intradermal challenge with *T*. *pallidum* Nichols (n = 3), SS14 (n = 3) or Mexico A (n = 3). None of the immune rabbit sera (IRS) tested recognized TP0751 (100 ng/strip), while all strongly recognized an equivalent amount of Tpp17 (**Fig 7A**). We next determined if humans with syphilis (n = 5) mount an antibody response to the protein. Sera from four patients with secondary syphilis failed to recognize the protein, while one reacted modestly: as with IRS, all patient sera strongly recognized Tpp17 and Tpp47 (**Fig 7B**). Since TP0751 is highly conserved among *T*. *pallidum* strains (**S7 Fig**), these results cannot be attributed to antigenic variation.

### TP0751 antibodies lack opsonic activity

Activity in the rabbit peritoneal macrophage opsonophagocytosis assay is considered an *ex vivo* surrogate for protective capacity [12, 90] as well as an indicator of surface exposure [16]. Houston *et al*. [43] reported that antiserum against TP0751 has opsonic activity greater than that of IRS. We found, however, that rabbit antisera directed against full-length TP0751 did not enhance uptake of spirochetes by rabbit peritoneal macrophages (**S8 Fig**). Consistent with previously reported results by immunofluorescence assay localizing Tpp17 to the periplasmic compartment of *T*. *pallidum* [37], Tpp17 antiserum also lacked opsonic activity.

### Immunization with TP0751^25-237^ does not protect rabbits from intradermal challenge

**Fig 8:**
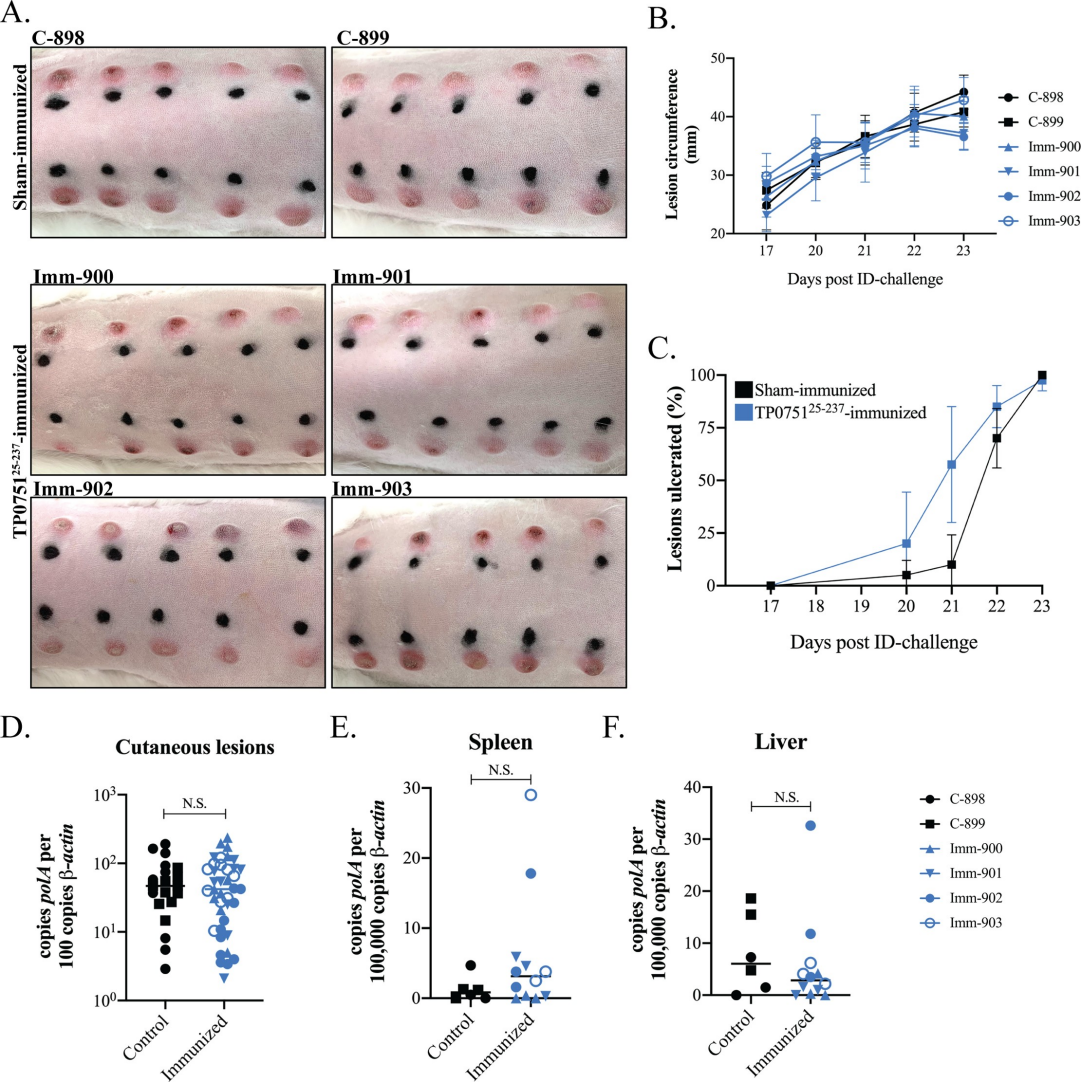
Immunization with TP0751^25-237^ does not attenuate lesion development or prevent dissemination of spirochetes. **(A)** Cutaneous lesions for TP0751^25-237^- and sham-immunized rabbits at sacrifice, 23 days post-challenge. **(B)** Average lesion circumference (mm) and **(C)** percentage of lesions ulcerating. No statistically significant differences were found in lesion circumference (nonlinear regression and extra sum of squares F-test) or lesion ulceration (multiple row *t* tests) between the two groups. Spirochete burdens in **(D)** cutaneous lesions, **(E)** spleens and **(F)** livers at the time of sacrifice (23 days post-challenge) assessed by qPCR (*polA* transcripts normalized to *β-actin*). Bars indicate the means of four TP0751-immunized and two sham-immunized animals. *T*. *pallidum* burdens in a punch biopsy of each cutaneious lesion and triplicate samples of liver and spleen from each rabbit were assayed by qPCR. *p* values for pairwise comparisons were determined using a two-tailed *t* test. (N.S. = not significant).

Lithgow *et al*. [47] reported that immunization of rabbits with TP0751 attenuated lesion development following intradermal challenge and inhibited dissemination of spirochetes. We sought to reproduce these findings following their immunization protocol with intact, full-length protein (TP0751^25-237^) (**S9A Fig**). By ELISA, all four immunized animals produced TP0751-specific antibodies with titers >1:200,000 (**S9B Fig**). In addition, immunoblots confirmed reactivity against ≤10 ng quantities of TP0751^25-237^ as well as the protein’s N- and C-terminal domains (TP0751^25-96^ and TP0751^97-237^) (**S9C Fig**). As expected, the two sham-immunized rabbits were non-reactive to TP0751 by ELISA and immunoblot (**S9B** and **S9C Fig**). Three weeks following the final boost, animals were challenged intradermally on their shaved backs with freshly-harvested *T*. *pallidum* (1 x 10^4^ organisms per site, 10 sites per animal). Erythema was observed at all sites by day 15 post-challenge. On day 17, motile treponemes were observed by darkfield (DF) microscopy in aspirates from two nodules per rabbit from each group (**S10A Fig**). Between days 17 and 23 post-challenge, we observed no difference in average lesion circumference (**Fig 8A** and **8B**) or percentage of lesions ulcerated (**Fig 8A**–**8C**). Following sacrifice on day 23, punch biopsies from all lesions were DF positive (**S3 Table**), and spirochete burdens were comparable by qPCR (**Fig 8D**). All lesions contained lymphoplasmacytic infiltrates characteristic of syphilis [91] with no discernible differences in degree of edema, infiltrate intensities, or relative proportions of lymphocytes, histiocytes, and plasma cells (**S4 Table**).

Although we were unable to detect *T*. *pallidum* DNA in blood from either group by qPCR (*polA* CT value > 40 cycles), comparable spirochete burdens were detected in the spleens and livers in both groups (**Fig 8E** and **8F**). To assess dissemination of live *T*. *pallidum*, popliteal lymph nodes (PLNs) from each challenged rabbit were transferred into a naïve animal (**S9A Fig.**). One recipient of PLNs (recipient rabbit #381) from a TP0751-immunized rabbit (Imm-900) developed a firm orchitis at day 41 post transfer and seroconverted by day 45 (**S5 Table**); of note, Imm-900 had one of the highest TP0751 antibody titers by ELISA and very strong immunoblot reactivity (**S9B** and **S9C Fig**). A recipient (#386) of PLNs from a control animal (C-899) developed orchitis on day 43 post transfer and seroconverted by day 45 (**S5 Table**). DF microscopy of the testicular extracts from recipients #381 and #386 revealed motile treponemes (**S5 Table** and **S10B Fig**). An immense cluster of motile organisms was observed in the extract from rabbit #381 (**S1 Video**). The other recipients failed to develop orchitis, did not seroconvert, and had negative DF and qPCR testicular exudates 90 days post transfer.

## Discussion

Efforts to deconvolute the complex pathogenesis of syphilis have long centered on the unusual OM of *T*. *pallidum* and the enigmatic surface it presents to its obligate human host [91, 92]. The dramatic global increase in the incidence of syphilis in the new millennium has created an urgent need for an effective vaccine [6] and, with it, an increased appreciation of the importance of *T*. *pallidum* OM ultrastructure and composition as the basis for selection of candidate vaccinogens [16]. In marked contrast to the OMs of Gram-negative bacteria, the *T*. *pallidum* OM is fragile, poorly antigenic, lacks lipopolysaccharide, and has a remarkably low density of integral OMPs [4, 16, 18, 19]. In recent years, great progress has been made in characterizing syphilis spirochete’s repertoire of rare, β-barrel-forming OMPs [16, 93–95]. The preponderance of *T*. *pallidum-*specific antibodies generated during human infection are directed against lipoproteins, not integral OMPs [3, 21, 23, 24]. Over the years many investigators in the syphilis field have conflated immunogenicity with surface exposure [16]. A robust body of microscopic [18, 37, 38], biochemical [87, 96], and structural evidence [88, 97] indicates that many *T*. *pallidum* lipoprotein immunogens are periplasmic, tethered by N-terminal lipids to the outer leaflet of the CM or the inner leaflet of the OM. Whether any lipoproteins reach the surface of the bacterium remains a subject of debate. It is noteworthy that the *T*. *pallidum* genome does not encode any secretory machinery other than the Sec translocon for export of proteins across the CM [19, 98] or the recently described OMP, SLAM, that transports lipoproteins to the surface of some Gram-negatives [99, 100].

The case for surface exposure of TP0751 rests largely on heterologous expression in *B*. *burgdorferi* [45, 46], the opsonic activity of TP0751 antibodies with rabbit peritoneal macrophages [43], and the protection reported for rabbits hyperimmunized with the protein [47]. Unlike *T*. *pallidum*, *B*. *burgdorferi* differentially expresses numerous lipoproteins on its surface [101, 102], indicating a facile lipoprotein secretion pathway clearly not present in the distantly related syphilis spirochete. Whether the *B*. *burgdorferi* ‘secreton’ [103] can distinguish between known periplasmic and putative surface lipoproteins of *T*. *pallidum* has not been rigorously established. Mislocalization of *T*. *pallidum* periplasmic proteins to the borrelial surface could give rise to interactions not reflective of those that occur during the disease process. Although heterologously expressed TP0751 was purportedly surface localized by flow cytometry, interpretation of these results is confounded by the inability to detect the protein by immunofluorescence or immunoblot analysis [45]. Houston *et al*. [43] reported that TP0751 antibodies promoted greater uptake of treponemes by rabbit peritoneal macrophages than immune rabbit serum, a finding we were unable to reproduce. Despite extraordinarily high titers of antibodies capable of detecting low nanogram quantities of the protein, in our hands, immunization failed to protect against local and disseminated infection following intradermal challenge. These considerations lead us to question the assertion that TP0751 promotes virulence from the *T*. *pallidum* surface.

To circumvent the caveats associated with surface localization methodologies in *T*. *pallidum* [16], we turned our attention to TP0751’s lipocalin domain. We took a structural approach given the finding by us and others [67, 68] that lipocalins are so diverse at the amino acid level that one cannot infer functional and evolutionary relationships from sequence comparisons. Rather than being ‘noncanonical’ [45], our examination of the TP0751^97-228^ structure revealed hallmark features of calycins and lipocalins. Results from DALI searches strongly reinforced the similarity between TP0751 and other members of the calycin superfamily by identifying more than 50 related structures of both eukaryotic and prokaryotic origin; this result clearly demonstrates that the lipocalin fold of TP0751 falls well within the broad evolutionary tapestry of the calycins. The absence of surface lipoproteins among the prokaryotic lipocalins is noteworthy. The structural similarity between factor H binding protein (fHbp), a surface lipoprotein of *Neisseria meningitides* [104], and TP0751 noted by Parker *et al*. [45] did not come up in our interrogation of the PDB database. Of the five studied bacterial structural orthologs from our search, three are periplasmic proteins from Gram-negatives. A fourth, from *B*. *subtilis* [73], demonstrates that a bacterium need not have an OM to harbor an acylated lipocalin; from an export standpoint, the surface of a Gram-positive bacterium is equivalent to the periplasm of a Gram-negative. The relatedness of Tpp17, the fifth ‘annotated’ bacterial lipocalin ortholog, to the N-terminal domain of the *E*. *coli* periplasmic lipoprotein NlpE already has been noted [39], but its lipocalin fold and structural similarity to TP0751 have not. Although the periplasmic location of Tpp17 recently has been called into question [35], we showed years ago using our gel microdroplet system that this lipoprotein is accessible to antibodies only in spirochetes whose OMs had been removed [37]. The structural inter-relationships revealed herein further support our assignment of Tpp17 and, by extension, TP0751 to the periplasmic compartment.

While the cavity of the TP0751 barrel is too small to accommodate hydrophobic compounds, *in silico* docking experiments provided strong proof of principle for ligand binding in a manner also characteristic of lipocalins—within the open end of the barrel [49, 75, 76]. Furthermore, the docking data predicted that the barrel rim contains regional distributions of amino acids that permit binding of multiple ligands in different configurations, a property also well described for some lipocalins [105, 106]. The combined *in silico* and binding results for heme seem a good ‘fit’ between *T*. *pallidum*’s minimalist requirements for iron [4] and the low expression levels of TP0751. Both iron and heme have inherent toxicities [107, 108], so acquisition mechanisms must carefully calibrate uptake with the cell’s homeostatic needs; low expression of TP0751, therefore, also would be inherently protective. In the absence of TonB-dependent uptake pathways, the mechanism(s) for scavenging of heme by *T*. *pallidum* and transport across the OM are unclear; this enigma is part of the broader conundrum of transition metal acquisition by the syphilis spirochete [4]. In any event, while the small molecule binding properties of TP751 require more investigation, the results presented herein provide proof of principle for a biologically plausible alternative to the atypical binding sites on the external façade of the lipocalin barrel proposed from peptide mapping [45, 74]. The structural similarities between the TP0751 lipocalin domain and Tpp17 are intriguing given the marked difference in their levels of expression. Close examination, however, reveals differences in their barrel rims that could influence their ligand preferences and binding properties. Tpp17 also has the ability to form disulfide bonded oligomers that can expand the accessible surface area for ligand interactions [39].

Deducing the function of a bacterial protein is best done with knowledge of its complete structure. Prior efforts to determine the structure of full-length TP0751 were unsuccessful because of degradation [42, 45]. However, the stability of TP0751^25-237^ in our hands enabled us to obtain a molecular envelope for the entire solvated polypeptide using SAXS. By combining SAXS with computational modeling, we determined that TP0751 has a dynamic, dual domain architecture consisting of an N-terminal IDR and a rigid, folded C-terminal lipocalin. IDRs act as autonomous units that transition through an ensemble of flexible conformations. Guided by short molecular recognition motifs within the unstructured domain, IDR-containing proteins ‘find’ their specific partner(s) and then fold to a stable conformation (‘coupled folding and binding’) [109, 110]. Once thought to be rare in bacteria [111], genomic surveys indicate that proteins with IDRs are roughly as common in prokaryotes as in eukaryotes [112]. In bacteria, IDRs often mediate protein-protein interactions within the periplasm [113–116]. By integrating structural and docking data, we envision an alternative functional scenario for TP0751. According to our conception, the unstructured IDR enables the polypeptide to probe or sweep its neighborhood within the periplasm, maximizing the likelihood of encountering a ligand for the lipocalin domain and/or binding partner(s). TP0751 has been previously described as a metalloprotein [43]; metal binding by the histidine-rich (seven His residues) IDR might play a regulatory role in these events. One obvious partner for the IDR is TP0750, a periplasmic non-lipoprotein which contains a Rossmann protein-protein interaction fold [117] and has been shown to bind TP0751 *in vitro* [44]. *In vivo* interaction between these two proteins requires residence in the same cellular compartment.

After a protracted struggle, the immune system of a patient with untreated syphilis eventually gains the upper hand over the ‘stealth pathogen’ [3, 4], driving down burdens and containing, if not eliminating, the bacterium [2]. It is widely believed that the appearance of opsonic antibodies, which promote the internalization, killing and degradation of this extracellular bacterium within phagolysosomes, marks a turning point in this battle [12–15]. A time-honored approach to vaccine development (“learning from nature”) has been to identify the targets of opsonic antibodies in syphilitic sera. Based on our observation that TP0751 elicits a barely detectable humoral response in infected rabbits and humans, it seems unlikely that anti-TP0751 antibodies contribute to the opsonic activity of syphilitic sera and, by extension, clearance of spirochetes. The poor antibody response probably reflects low expression of native protein by spirochetes during the course of infection. It is, of course, possible for a protein to be poorly antigenic during infection but still protective by hyper-immunization. We did not find this to be the case either, despite inoculating each site with a 100-fold lower dose of treponemes (1 x 10^4^ vs 1 x 10^6^) than used previously [47] to ensure we did not miss a protective response. The only evidence of protection was recovery of treponemes by PLN transfer from one of four immunized rabbits as opposed to one of two sham-immunized controls. We attribute this to biological variability since spirochete burdens in livers and spleens were no different in the two groups, and PLNs from a TP0751^25-237^ immunized rabbit (titer > 1:200,000) caused a florid orchitis in a recipient animal. The inability of TP0751 to elicit opsonic or protective antibodies is consistent with a subsurface location.

## Material and methods

### Ethics statement

Enrollment of individuals with secondary syphilis was carried out in accordance with the recommendations of the Institutional Review Board at Centro Internacional de Entrenamiento e Investigaciones Médicas (CIDEIM) in Cali, Colombia. All animal experimentation was conducted following the *Guide for the Care and Use of Laboratory Animals* (8th Edition) and in accordance with protocols reviewed and approved by the UConn Health Institutional Animal Care and Use Committee under the auspices of Animal Welfare Assurance A3471-01.

### Human secondary syphilis sera

Adult HIV-seronegative secondary syphilis (SS) patients were enrolled through a previously described network of health care professionals in Cali, Colombia [13, 118]. The diagnosis of SS was based on the medical history and compatible skin or mucosal lesions, a rapid plasma reagin (RPR) titer ≥1:8 and a positive rapid treponemal test. Phlebotomy was performed following informed consent.

### Propagation of *T*. *pallidum* strains and generation of immune rabbit sera

The Nichols, SS14 and Mexico A strains of *T*. *pallidum* were propagated by intratesticular inoculation of adult male New Zealand White (NZW) rabbits and harvested in CMRL medium (Gibco) supplemented with 20% heat-inactivated normal rabbit sera (NRS) at peak orchitis. Spirochetes were enumerated by DF microscopy on a Petroff-Hausser counting chamber (Hausser Scientific). Immune rabbit sera (IRS) for each strain were generated by intratesticular inoculation of three nonreactive adult male NZW rabbits with 1x10^7^ treponemes in 500 μl CMRL containing 20% NRS per testis. Sixty days post-inoculation, animals were intradermally challenged with 1 x 10^3^ freshly extracted treponemes of the homologous strain at each of 8 sites on their shaved backs. Animals were euthanized and exsanguinated 30 days later once their immune status had been confirmed by lack of lesion development.

### Cloning procedures

DNA encoding full-length TP0751 without its signal sequence (TP0751^25-237^, amino acid residues 25 through 237); Accession number: WP_010882196) was PCR-amplified from *T*. *pallidum* genomic DNA (gDNA) (see **S2 Table** for primers); the resulting amplicon was cloned into the BamHI (5′-end) and Xho1 (3′-end) sites of pET-23b (Novagen) in-frame with the C-terminal His_6_-tag. DNAs encoding the N-terminal IDR (TP0751^25-96^) and C-terminal lipocalin domains of TP0751 (TP0751^*97-237*^) were amplified from the pET-23b plasmid harboring *tp0751*^*25-237*^. By using the Inverse In-fusion PCR cloning (IFPC) method [119] (Takara), the resulting amplicons were cloned into the vector coordinates of NheI (5′-end) and XhoI (3′-end) restriction sites of pET-28a in-frame with the N-terminal His_6_-tag. DNA encoding TP0750 without its signal sequence (residues 22–224, Accession number: WP_010882195) was PCR-amplified from *T*. *pallidum* DNA. The resulting amplicon was cloned into the NheI (5′-end) and XhoI (3′-end) restriction sites of pET-28a in-frame with the N-terminal his_6_-tag using the IFPC method [119]. DNAs encoding Tpp17 (TP0435, Accession number: WP_010881883) and Tpp47 (TP0574, Accession number: WP_010882021) without signal sequences were PCR-amplified from *T*. *pallidum* gDNA. The amplicon for *tp0435* was cloned into the BamH1 (5′-end) and EcoRI (3′-end) restriction sites of pET-23b in-frame with the C-terminal His_6_-tag. The amplicon for *tpp47* was cloned into the BamH1 (5′-end) and SalI (3′-end) restriction sites of pET-23b in-frame with the C-terminal His_6_-tag. All of the above constructs were confirmed by Sanger sequencing (Genewiz, Inc.).

### Expression and purification

All constructs used in this study were expressed in *E*. *coli* Overexpress C41 (DE3) cells (Lucigen Corporation). Cultures were grown in LB-Miller broth containing the appropriate antibiotics (50 μg/ml) at 37°C with shaking. Expression was induced at A_600_ = ∼0.4 by the addition of isopropyl β-d-1-thiogalactopyranoside (IPTG) to a final concentration of 0.5 mM, followed by 3–4 h of growth. Cells were harvested by centrifugation at 6000 × *g* for 15 min at 4°C. The resulting cell pellets were resuspended in 40 ml of lysis buffer consisting of 50 mM Tris (pH 7.5), 100 mM NaCl, 10% glycerol, and 100μl of protease inhibitor cocktail (Roche Diagnostics). The cell lysates were centrifuged at 20,000 × *g* for 30 min at 4°C and loaded onto a 5 ml Ni–NTA (Qiagen) column pre-equilibrated with Buffer A consisting of 50 mM Tris (pH 7.5), 100 mM NaCl, 10 mM imidazole and 1 mM β-mercaptoethanol (BME). The proteins were eluted from the Ni-NTA resin with Buffer A supplemented with 300 mM imidazole after thorough washing with Buffer B [50 mM Tris (pH 7.5), 200 mM NaCl, 10 mM imidazole]. Elution fractions were pooled and subjected to size-exclusion chromatography using a Sephacryl S-200 HR 16/60 column (GE Healthcare Life Sciences) pre-equilibrated with 50 mM Tris (pH 7.5), 100 mM NaCl and 1 mM dithiothreitol (DTT). The purity of all proteins was verified by sodium dodecyl sulfate-polyacrylamide gel electrophoresis (SDS-PAGE). For rabbit protection experiments (see below), intact, purified TP0751^25-237^ was concentrated to 6 mg/ml using an Amicon filtration device (Millipore), flash-cooled in liquid nitrogen, and stored at −80°C (see below).

### Analytical gel-filtration

Gel filtration experiments were carried out using a Superdex 200 Increase 10/300 GL column (GE Healthcare) and a DuoFlow chromatography system (Bio-Rad). The column was calibrated using thyroglobulin (670 Da), ferritin (440 kDa), aldolase (158 Da), conalbumin (75 kDa), chymotrypsinogen (25 kDa), and ribonuclease A (13.7 kDa). The standard curve relating relative elution volumes to molecular weights was calculated using Prism (Graph Pad) by regressing log molecular weights as a function of partition coefficients (*K*_*av*_) using the equation *K*_*av*_ *= Ve–V*_*0*_ */ V*_*g*_*−V*_*0*_, where *Ve* is the protein elution volume, *V*_*0*_ is the void volume determined by elution of Blue Dextran (2000 kDa), and *Vg* is the geometric column volume. A total of 500 μl of sample was loaded on the column pre-equilibrated in buffer containing 50 mM Tris–HCl (pH 7.5), 50 mM NaCl and 1 mm DTT and eluted using the same buffer at a flow rate of 0.5 ml/min, with detection at 280 nm.

### Small-angle X-ray scattering (SAXS) data acquisition and analysis

SAXS data for TP0751^24-237^ samples containing 1.0 mg/ml of protein were collected at beamline 16-ID (LiX) of the National Synchrotron Light Source II (Upton, NY) using a wavelength of 0.82 Å in a three-camera configuration for 10 consecutive 2-s exposures. Yielded accessible scattering angles were 0.006 < q < 3.0 Å^-1^ where q is the momentum transfer, defined as q = 4π sin(θ)/ λ, where λ is the Xray wavelength, and 2θ is the scattering angle; data to q<0.5Å were used in subsequent analysis. The data processing program pyXS [120] was used for scaling, integration, and averaging of individual scattering images after inspection for aggregation. Radii of gyration (R_g_), deduced from the Guinier region of the Guinier plots, were computed using PRIMUS [121]. P(*r*) functions were calculated using GNOM [122]. Theoretical scattering curves were compared against experimental scattering data using the program FoXS [123]. Normal mode analysis (NMA) was performed using the program Sreflex [124] built into the ATSAS software package [125]. *Ab initio* envelope reconstructions were performed using DAMMIN [61] and DAMAVER [62].

### Protein modeling and bioinformatics analysis

To facilitate interpretation of the SAXS data, a structural model of TP0751^25-237^ was generated using the “Intensive mode of modeling” module in the Phyre2 server (http://www.sbg.bio.ic.ac.uk/phyre2/html/page.cgi?id=index) [56]. The atomic file (PDB ID: 2JK2-F) of the TP0751^97-228^ crystal structure was submitted to the DALI server (http://ekhidna2.biocenter.helsinki.fi/dali/) [71] to find orthologs in the RCSB PDB structural database (http://www.rcsb.org). Structures were aligned using the SSM superimpose algorithm implemented in WinCoot [126]. Topology diagrams for TP0751^25-237^ and Tpp17 were generated using PDBsum [127]. All structural figures were rendered in PyMol (https://pymol.org/) [128]. Structure-based sequence alignment was done using PROMALS3D [129] and ESript3.0 [130]

### Calculation of disordered regions in TP0751

Disordered regions of TP0751^25-237^ were calculated using IUPred2 (https://iupred2a.elte.hu/) [58], PrDOS (http://prdos.hgc.jp/cgi-bin/top.cgi) [57], PONDR (http://www.pondr.com/) [131].

### Automated ligand search

The atomic file (PDB ID: 2JK2-F) of the TP0751^97-228^ was submitted to the COACH [77, 78] and 3DLigandSite [79] servers. The output PDB files of the TP0751-ligand complex were analyzed in PyMol (https://pymol.org/).

### *In silico* docking

PDBs of candidate ligands were extracted from the RCSB PDB structural database. We used AutoDock 4.2 [81] to estimate free energies of ligand binding. Rigid-body docking was performed using a grid box size of 50 × 50 × 50 Å along the *X*, *Y*, and *Z* axes and centered at coordinates (72.769 49.091 34.004) around PDB of TP0751^97-228^ (**S3 Fig**). Docking simulations were performed for 100 runs using a Lamarckian Genetic Algorithm (LGA) [81]. The results were evaluated using RMSD values, ligand-protein interactions, and binding energies (ΔG_bind_). For each ligand, the protein-ligand complexes with the lowest energy were analyzed using Ligplot+ [84].

### Heme binding assay

Heme binding was assayed as described by Peherstorfer *et al*., [132]. Hemin (Sigma) was dissolved in 30 mM NaOH to a concentration of 1 mM and incubated in the dark on ice for 30 min. Stock solutions were diluted in HEPES buffer (100 mM, pH 7.0) to the desired concentration directly before use. Heme concentrations in the dilutions were determined using the molar extinction coefficient of heme (ε^385^ = 58.440M^−1^ cm^−1^). A SYNERGY H1 spectrophotometer (BioRek) was used for UV/Vis measurements to detect heme binding. Lysozyme (Sigma) was dissolved in 50 mM HEPES buffer (pH 7.0). Heme (0–30 μM) was mixed with 5 μM of purified TP0751^25-96^ or TP0751^97-237^ or 10 μM lysozyme as a negative control [85] and incubated in the dark at room temperature for 60 min to ensure complete complex formation before UV/Vis spectra were measured at 300 to 600 nm. For each measurement, the absorbance intensity was corrected by subtracting the absorbance of the sample containing only heme. Then, the subtracted absorbance at 416 nm was plotted against the total concentration of heme. The apparent *K*_d_ was estimated by fitting the data to the following equation (one-site binding with the Hill slope):

$$\theta ={\left[L\right]}^{n}/{\left[L\right]}^{n}+K_{d}$$

were θ is the fraction of the protein concentration bound by the ligand, [L] is the concentration of unbound ligand, and n is the Hill coefficient.

### Measurement of *tp0750* and *tp0751* transcripts by qRT-PCR

Total RNAs were extracted from the testicular tissue of four rabbits inoculated with *T*. *pallidum*, Nichols strain, according to our prior publications [133]. Concentrations were determined using a Nanodrop spectrophotometer (Thermo-Scientific). cDNA synthesis was performed using the High Capacity cDNA Archive Kit (Applied Biosystems) according to the manufacturer’s instructions. Primers (**S2 Table**) were used to amply transcripts for *flaA* (*tp0249)*, *tp0435*, *tp0750*, *tp0751* in addition to the intergenic region between *tp0750* and *tp0751*. Thermocycling was performed in a GeneAmp PCR System 9700 (Applied Biosystems) as follows: one cycle at 95°C for 2 min, followed by 35 cycles of 98°C for 10 sec, 55°C for 15 sec 72°C for 15 sec, and one cycle of 72°C for 15 sec. Following amplification, six μl of each gel-purified PCR product were poly-A tailed using TaqEx DNA polymerase kit (Takara) and dATP according to manufacturing recommendations. Thermocycling was performed as follows: 1 cycle at 70°C for 20 min. Four μl of each gel-purified PCR product was cloned using a TOPO TA Cloning Kit, with an Xhol enzyme digested pCR2.1-TOPO plasmid (Thermofisher Scientific) according to manufacturer’s direction and then transformed into Top10 *E*. *coli* cells. Plasmid from three individual colonies was isolated using a QIAprep Spin Miniprep Kit and sequences were confirmed by Sanger sequencing. Standard curves were created for *flaA*, *tp0435*, *tp0750*, *tp0751* and the *tp0750*-*tp0751* intergenic region using 10-fold serial dilutions from 1 x 10^7^ to 10^2^ copies of plasmid TOPO clone standards. Amplification reactions were performed in a Bio-Rad CFX96 Real-Time PCR Detection System using the following conditions: 95°C for 2 min, and 35 cycles of 95°C for 10 s, 57°C for 15 s and 72°C for 30 s followed by 72°C for 5 min. All assays were confirmed to have efficiency and R^2^ values greater than 90% and 0.95, respectively. All samples and standards were run as 25 μl reactions in 0.2 ml PCR strip tubes with optically clear caps in triplicate and each PCR run included negative (no template) control reactions. Expression levels for all transcripts were normalized to copies of *flaA*.

### Generation of antisera

Rat polyclonal antiserum directed against Tpp17, Tpp47, TP0750 and TP0751^25-237^ were generated in 6-week-old female Sprague-Dawley rats as described previously [95]. Rat antiserum to p30.5 (TP0453) was described previously [89]. For opsonophagocytosis assays (described below), rabbit polyclonal antisera directed against Tpp17, and TP0751^25-237^ were generated in rabbits by Rockland, Inc., according to their established protocol.

### Opsonophagocytosis assay

Opsonophagocytosis assays were performed as described previously [134, 135]. Rabbit peritoneal macrophages were elicited by intraperitoneal injection of 10 ml of 15% sterile Proteose Peptone no. 3 (Sigma-Aldrich). Cells were harvested 4 days later by peritoneal lavage with PBS containing 10 U of heparin (Sigma-Aldrich) per ml, centrifuged at 900 × *g* for 10 min, and resuspended in DMEM supplemented with 10% fetal bovine serum (FBS). The macrophages then were counted with a hemocytometer and plated in BioCoat poly-D-lysine-treated culture slides at a density of 5 × 10^5^ cells/ml. After incubation for 2 h at 37°C, nonadherent cells were removed by washing the monolayers twice with DMEM. The cells were maintained overnight at 37°C and 5% CO_2_. The following day, the adherent macrophages were washed twice with DMEM. Freshly harvested *T*. *pallidum*, adjusted to a final concentration of 3 x 10^8^ treponemes per ml, were incubated with either 10% NRS, IRS, anti-Tpp17 or anti-TP0751^25-237^ serum for 2 h at RT prior to addition to rabbit peritoneal macrophages. *T*. *pallidum* were added at a multiplicity of infection (MOI) of 10:1 for 4 h at 37°C with 5% CO_2_. Following incubation, supernatants were removed, and macrophages were prepared for IFA to evaluate uptake of treponemes. Cells were fixed and permeabilized with 2% paraformaldehyde (PFA) and 0.01% Triton X-100 for 10 mins at RT. They then were rinsed with PBS, blocked with PBS containing 10% normal goat serum (NGS) for 1 h at RT, and then incubated with rat polyclonal anti-FlaA [136] (1:100) in PBS 1% NGS for 1 h at RT. After four successive washes with PBS, the cells were then incubated with goat anti-rat IgG-AF488 (1:500) in PBS 1% NGS for 1 h at RT. After staining for *T*. *pallidum*, the cells were then washed thoroughly with PBS 6 times, rinsed with deionized (DI) water to remove salt and allowed to air dry. Finally, Vectashield containing DAPI (Vector Laboratories, Inc.) was added and samples were sealed with a coverslip. To assess the internalization of *T*. *pallidum*, images of 100 macrophages were acquired on an Olympus BX60 epifluorescence microscope equipped with a Retiga 2000R camera (QImaging). Acquired images were processed with ImageJ (version 1.5.1g) (NIH) and uptake was quantitated in a blinded fashion. The percentage of spirochete-positive macrophages was calculated by dividing the number of cells containing ≥1 internalized spirochetes by the total number of cells imaged.

### SDS-PAGE and immunoblotting

To detect TP0750 and TP0751 in *T*. *pallidum* lysates, MARBLOT strips (Trinity Biotech) were blocked for 1 h with PBS, 5% nonfat dry milk, and 0.1% Tween 20 and then probed overnight at 4°C with antisera against TP0750, TP0751^25-237^, Tpp17, p30.5, Tpp47, and NRS at a concentration of 1:1,000. After being washed with PBS and 0.05% Tween 20 (PBST), the membranes were incubated for 1 h at RT with HRP-conjugated goat anti-rat IgG antibodies at a concentration of 1:30,000. Following washes with PBST, the immunoblot strips were lined up and developed on a single film using the SuperSignal West Pico chemiluminescent substrate.

To detect antibodies generated during human infection, recombinant 100 ng of Tpp17, Tpp47 and TP0751^25-237^ were resolved by SDS-PAGE using Any kD Mini-Protean TGX gels (Bio-Rad) and transferred to nitrocellulose membranes (0.45-μm pore size; GE Healthcare) at 15 V for 15 min using a semidry apparatus (Bio-Rad). Membranes were blocked for 1 h with PBS, 5% nonfat dry milk, and 0.1% Tween 20 and probed overnight at 4°C with a dilution of 1:250 of NRS, IRS, normal human serum (NHS, (Corning)) and serum from five individuals diagnosed with secondary syphilis described above. After being washed with PBST, the membranes were incubated for 1 h at RT with HRP-conjugated goat anti-rabbit IgG or goat anti-human IgG antibodies at a dilution of 1:30,000. Following washes with PBST, the immunoblots were developed using the SuperSignal West Pico chemiluminescent substrate.

To detect antibody reactivity against full-length TP0751 and its two domains, graded amounts (1 to 100 ng) of TP0751^25-237^, TP0751^25-96^ and TP0751^97-237^ were resolved by SDS-PAGE and transferred to nitrocellulose membranes. The membranes were probed overnight at 4°C with 1:1000 dilutions of sera from TP0751^25-237^- and sham-immunized rabbits. After washing with PBST, the membranes were incubated for 1 h at RT with a 1:30,000 dilution of HRP-conjugated goat anti-rabbit IgG antibody. Following a second wash with PBST, immunoblots were developed using the SuperSignal West Pico chemiluminescent substrate.

### Calculation of conservation score for TP0751

An alignment of TP0751^25-237^ sequences from 32 different strains of *T*. *pallidum* (**S6 Table)** was generated with Clustal Omega [137]. The conservation score for each position of TP0751 was calculated and superimposed on the structural model of TP0751^25-237^ using the Shannon entropy analysis of the protein variability server (PVS) [138].

### Immunization of rabbits with TP0751^25-237^

Following the protocol described by Lithgow *et al*. [47], a cohort of six male NZW rabbits (~3.5kg, 13–15 weeks of age, Covance) with RPR and TP-PA negative serologies were divided randomly into two groups: TP0751^25-237^-immunized (n = 4) and sham-immunized controls (n = 2). Rabbits were sedated and immunized a total of three times with a 1:1 mixture of the TP0751 full-length high-quality recombinant protein (0.52 mg per ml) and TiterMax Gold Adjuvant. Each immunization was delivered as four subcutaneous (0.1 μl per site) injections in the shoulder regions and two intramuscular (0.04 μl per site) injections in the quadricep muscle. Booster immunizations with identical doses were administered at 3 and 6 weeks following the primary injections. Sham controls received PBS alone with the TiterMax Gold Adjuvant at corresponding time points.

### Quantitation by ELISA of TP0751 antibodies in immunized rabbits

Antibody titers against Tp0751^25-237^ were determined by ELISA. Clear flat-bottom Immuno nonsterile 96-well plates (Thermo Scientific) were coated with 100 ng of recombinant TP0751^25-237^ overnight at 4°C Plates were washed with PBST and blocked with 1% bovine serum albumin (BSA) in PBS for 1 h at RT. Serum from each rabbit was serially diluted from a starting concentration of 1:100 to a final concentration of 1:200,000 in PBS containing 1% BSA. Fifty μl of each sample concentration was added to the ELISA plate in duplicate. After 2 h of incubation at RT, the plates were washed three times with PBST, followed by the addition of 50 μl of HRP-conjugated goat anti-rabbit IgG antibody diluted 1:10,000 in PBS with 1% BSA to each well. After 2 h of incubation at RT, the plates were washed five times with PBST, 100 μl of TMB chromogen substrate solution (Invitrogen Novex) was added to each well followed by incubation for 15 min at RT. One hundred μl of Stop solution (Invitrogen) was added and absorbance measurements (450-nm wavelength) were taken. After subtraction of background (no protein) values, means ± standard errors of the means (SEMs) of results from duplicate wells were calculated.

### Assessment of protective capacity of TP0751

TP0751^25-237^- and sham-immunized animals were challenged at each of 10 sites on their shaved backs with 1 x 10^4^ freshly harvested treponemes in 100 CMRL with 20% NRS; a single 1 ml syringe containing 600 μl of 1 x 10^5^ *T*. *pallidum* per ml was used per side of each animal. Animals were examined daily to monitor the development, morphologic appearance, and progression of lesions. On day 17 post-challenge, aspirates were obtained from two nodular lesions per rabbit using a 26-gauge needle and 0.9% sterile saline and examined in a blinded manner for motile treponemes using an Olympus BX51 microscope equipped with a DF condenser and Retiga R6 camera (Ocular version 2.0). Lesions were measured daily with digital calipers from day 17 post-challenge until sacrifice on day 23. On days 12, 17 and 23 post-challenge, fresh blood was collected into an EDTA tube and immediately aliquoted into 1 ml volumes for DNA extraction and *polA* qPCR (detailed below). Following euthanasia, each cutaneous lesion was excised and subdivided using a sterile 4-mm punch for DF microscopy, histopathology and qPCR. Punch biopsies for DF microscopy were placed in separate 1.8 ml Eppendorf tubes containing 250 μl of Dulbecco Modified Eagle Medium (DMEM) supplemented with 20% NRS. For each sample, findings from four fields were scored in a blinded fashion as + (1–5 *T*. *pallidum* per field), ++ (6–10 *T*. *pallidum* per field), +++ (>10 *T*. *pallidum* per field). A second punch biopsy was fixed in 10% buffered formalin and prepared for histopathologic analysis; three hematoxylin and eosin stained sections from each sample were reviewed by a pathologist (MF) in a blinded manner. The third skin biopsy, and three samples of liver and spleen from each rabbit, were placed in 1.8 ml Eppendorf tubes containing 500 μl of Zymo DNA/RNA Shield and stored at -20°C until extraction of DNAs.

### DNA extraction from *T*. *pallidum* challenged rabbits

DNAs were extracted immediately from 1 ml aliquots of freshly collected blood using a Qiagen Blood Midi Kit according to the manufacturer's protocol. DNAs were eluted with 200 μl of elution buffer.

Prior to DNA extraction, samples of skin, spleen and liver were transferred to individual clean 1.8 ml tube and quickly centrifuged to remove the DNA protectant. DNA extraction was performed using a Qiagen DNeasy Blood and Tissue kit following the manufacturer’s protocol with minor modifications. Tissue samples were digested with 200 μl of 2% Collagenase Type IV in PBS for 4 h at 37°C. Two hundred μl of 2 mg/ml Proteinase K made up in PK Buffer (200 mM NaCl, 20 mM Tris-HCl (pH 8.0), 50 mM EDTA, and 1% SDS) was added to the digested tissues and incubated at 56°C overnight. 200 μl of the digested tissue, 200 μl of Qiagen DNeasy Blood & Tissue kit ATL buffer, and 20 μl of Proteinase K (20 mg/ml) were combined in a new tube and incubated at 56°C for 10 mins. DNA precipitation and column washes were in accordance with the manufacturer's protocol. DNA was eluted from the QIAGEN columns with 200 μl (skin lesions) or 100 μl (spleen and liver) of elution buffer.

### Assay of *T*. *pallidum* tissue burdens by qPCR

PCR amplification of the *b-actin* gene was performed using Applied Biosystems TaqMan primers (Oc06813675_s1 VIC_MGB, ThermoFisher) in 25 μl reaction containing 2.5 μl of rabbit gDNA, 2.5 μl 10X buffer, 2.5 μl dNTPs, 1.25 μl 20X primer/probe, 0.25 μl ExTaq enzyme and 16 μl of DNase-free water. Thermocycling was performed in a GeneAmp PCR System 9700 (Applied Biosystems) as follows: one cycle at 95°C for 10 min and 40 cycles of 95°C for 15 sec and 60°C for 1 min. Resulting PCR reactions were run on a 2% agarose gel, and bands were excised for purification using a Gel and PCR clean up kit (Macherey-Nagel) according to manufacturer’s instructions; amplicons were eluted in 15 μl of sterile DNase free water. To generate a *b-actin* qPCR standard, 4 μl of gel-purified PCR product was cloned using a TOPO TA Cloning Kit, with pCR2.1-TOPO (Thermofisher Scientific) according to manufacturer’s direction and then transformed into Top10 *E*. *coli* cells. Plasmid from three individual colonies was isolated using a QIAprep Spin Miniprep Kit and *b-actin* sequence was confirmed by Sanger sequencing. PCR amplification and generation of linearized plasmid standard of the *T*. *pallidum polA* gene was performed as previously published [13]. Quantification of *T*. *pallidum* in samples was achieved using *T*. *pallidum polA*-F and *polA*-R primers and a TaqMan probe (**S2 Table**). Standard curves were created for *polA* and *b-actin* using 10-fold serial dilutions from 1 x 10^6^ to 10^1^ copies of plasmid TOPO clone standards. All assays were confirmed to have efficiency and R^2^ values greater than 90% and 0.95, respectively. All samples and standards were run as 25 μl or 50 μl reactions in 0.2 ml PCR strip tubes with optically clear caps in triplicate and each PCR run included negative (no template) control reactions. Thermocycling was performed in a Bio-Rad CFX96 Real-Time PCR Detection System as follows: 1 cycle of 95°C for 10 min followed by 40 cycles of 95°C for 15 sec and 60°C for 1 min. The *T*. *pallidum* and *b-actin* copy numbers for each specimen were extrapolated from the standard curves generated using ten-fold serial dilutions of plasmid standards. The raw data obtained from the amplifications were adjusted for quantity tested to generate the number of copies of *polA* per number of copies of rabbit *b-actin*.

### Popliteal lymph node transfer

Following sacrifice, PLNs from each rabbit were excised and disrupted in a 6-well sterile plate with DMEM containing 20% NRS using a sterile cell strainer. The strained material from each rabbit then was injected into the testes of six individual, RPR non-reactive rabbits, as described previously by Lukehart *et al*. [139]. Beginning on day 7, animals were monitored every other day for signs of orchitis and, beginning on day 30, assessed every two weeks for seroconversion (RPR and TP-PA). Animals that showed signs of orchitis and seroconverted were euthanized, the testes were harvested and, the testicular exudates assessed by DF microscopy. Animals that had not developed orchitis or seroconverted day by 90 were sacrificed, testes were harvested and testicular exudates were assessed by DF microscopy and qPCR.

### Statistical analysis

Data from the qRT-PCR experiments were normalized as described above and a multiple comparisons Kruskal-Wallis test with a Dunn’s correction was used to determine statistical significance. Cutaneous lesion circumferences were assessed using nonlinear regression and extra sum of squares F-test and lesion ulceration between the groups were compared using multiple t-tests. *T*. *pallidum* burdens measured by qPCR were compared using a Student’s *t*-test. Data were graphed and statistical analyses were performed using Prism 8.0 (GraphPad Software).

## Supporting information

S1 Fig Full-length TP0751 is a stable monomer. **(A)** Purification of recombinant TP0751^25-237^ from *E*. *coli* under non-denaturing conditions using affinity chromatography. Lanes 1–6 represent molecular weight markers, supernatant of induced culture lysate, flow-through, 30 mM imidazole wash, 80 mM imidazole wash, and protein eluted in 300 mM imidazole from the nickel–nitrilotriacetic column, respectively. **(B)** Size-exclusion chromatography (SEC) of TP0751^25-237^ produces a single peak corresponding to a molecular weight of ~25 kDa. The inset shows the SEC calibration curve calculated by a linear fit of known molecular weight (M.W.) standards as a function of measured partition coefficients (K_av_). The red and black circles, respectively, show the partition coefficients of recombinant TP0751^25-237^ and calibration standards.
(TIF)

S2 Fig The overall architecture of TP0751^97-228^. The lipocalin fold of TP0751^97-228^ depicted as a ribbon model in which α helices are shown in red, β strands are shown in yellow, and loops are shown in green. The Ω-loop is shown in magenta. Residues of the calycin signature motif are outlined by the dashed oval and labeled. The arrow indicates a 3_10_-helix located on the closed side of the β barrel.
(TIF)

S3 Fig Grid box for TP0751^97-228^ docking calculations. Ligand-binding site in TP0751^97-228^ for **(A)** retinol and **(B)** heme predicted by COACH and 3DLigandSite. Residues of steric clashes are depicted as red sticks. **(C)** The AutoDock grid box (50 × 50 × 50 Å along the *X*, *Y*, and *Z* axes) used for docking calculations. The solid black box centered at coordinates *X*:72.769, *Y*:49.091, *Z*:34.004 (PDB ID: 5JK2) represents the coverage of the docking grid. Previously reported laminin-binding peptides [45] p4 (residues 97–111, cyan), p6 (residues 116–138, orange), p10 (residues 172–195, magenta) and p11 (residues 196–209, yellow) mapped onto the structure of TP0751^97-228^. p10 also has also been reported to interact with LamR [74]. TP0751^97-228^ is shown in a different orientation compared to other figures to facilitate presentation of the grid box and the locations of laminin-binding peptides. (**D)** Electrostatic potential of TP0751^97-228^. Dashed lines indicate the location of different peptides on the surface of the TP0751^97-228^ structure. Panels C and D are in the same orientation.
(TIF)

S4 Fig Structure-based sequence alignment between TP0751^97-228^ and Tpp17. Identical residues are highlighted in black. The secondary structure elements (α-helix: spiral line; β strand: arrow) are shown for both proteins above and below their respective sequences. Residues of the Ω-type loop are underlined.
(TIF)

S5 Fig Comparative electrostatics (same orientation) of TP0751^97-228^ (PDB ID: 5JK2), Tpp17 (PDB ID:4U3Q) and the N-terminal domain (residues 21–103) of *E*. *coli* NlpE (PDB ID:4Z4H). The surface is colored according to the local electrostatic potential (−5 kT to +5 kT), calculated using the ABPS plug in in PyMOL (https://pymol.org/). The open end of each β-barrel is indicated by an arrow in the front view. Dashed lines represent the exterior of barrel rim.
(TIF)

S6 Fig Rooted circular cladogram showing the sequence-based phylogenetic relationships between TP0751^97-226^ and the 15 bacterial lipocalins identified by the DALI survey as structural orthologs. PDB IDs of bacterial lipocalins and Z-scores against TP0751^97-226^ are in parentheses.
(TIF)

S7 Fig TP0751 is fully conserved in *T*. *pallidum*. **(A)** Multiple sequence alignment of TP0751^25-237^ in the Nichols, Mexico A and SS14 strains. (**B)** Conserved residues of TP0751^25-237^, defined by sequence alignments of 32 different strains of *T*. *pallidum*, superimposed on the homology model and colored according to Shannon entropy scores.
(TIF)

S8 Fig TP0751 antisera lacks opsonic activity for *T*. *pallidum*. Freshly extracted *T*. *pallidum* were incubated with 10% heat-inactivated normal rabbit serum (NRS), immune rabbit serum (IRS), or rabbit antisera to TP0751^25-237^ or Tpp17 for 2 h prior to incubation with rabbit peritoneal macrophages (PeriMΦ) for 4 h at an MOI 10:1. Following incubation, *T*. *pallidum* and nuclei were labeled as described in Section “Materials and Methods.” Spirochetal uptake was quantified as % *T*. *pallidum*^+^ *(Tp)* macrophages. The experiments were graphed individually.
(TIF)

S9 Fig Experimental timeline of immunization and challenge. **(A)** TP0751^25-237^- (n = 4) and sham-immunized (n = 2) rabbits were given four 0.1 ml subcutaneous injections into the shoulders and two 0.04 ml intramuscular injections of TP0751^25-237^ or PBS into the quadricep muscles containing recombinant TP0751 and buffer, respectively as described by Lithgow *et al*. [47]. Following immunization, animals were challenged by intradermal inoculation with 1 x 10^4^ freshly extracted *T*. *pallidum* at each of 10 sites on their shaved backs. On day 23 post-challenge, animals were euthanized, and organs were harvested for qPCR assessment of bacterial burden. Popliteal lymph nodes (PLNs) were injected into the testes of naïve animals and followed for 90 days. **(B)** Serum antibody titers determined by ELISA (100 ng of TP0751^25-237^ per well). **(C)** Reactivity of sera from immunized and control rabbits determined by immunoblot analysis against graded concentrations of TP0751^25-237^, TP0751^25-96^ and TP0751^97-237^.
(TIF)

S10 Fig Darkfield analysis of cutaneious lesions and testicular exudate. **(A)** Cutaneous lesions for TP0751^25-237^- and sham-immunized rabbits on day 17 post-challenge. Aspirates were collected from two sites (indicated asterisks) and assessed by DF microscopy (insets on right). **(B)** Representative DF micrographs (60X magnification) of the testicular exudate from rabbit #386 (sham-immunized) and #381 (TP0751^25-237^-immunized PLN recipient) on day 45 post-PLN transfer.
(TIF)

S1 Video Darkfield microscopy of testicular exudate from rabbit #381, a PLN recipient from a TP0751^25-237^-immunized rabbit. (MP4)

S1 Table TP0751 structural orthologs. (XLSX)

S2 Table Primers. (XLSX)

S3 Table Darkfield findings from cutaneous lesions. (XLSX)

S4 Table Histopathologic analysis of cutaneous lesions. (XLSX)

S5 Table PLN-transfer summary. (XLSX)

S6 Table *T*. *pallidum* strain list. (XLSX)

## References

[1] Hook EWR. Syphilis. Lancet. 2017;389(10078):1550–7. 10.1016/S0140-6736(16)32411-4 27993382

[2] Radolf JD, Tramont EC, Salazar JC. Syphilis (*Treponema pallidum*). In: Mandell GL, Dolin R, Blaser MJ, editors. Mandell, Douglas and Bennett's Principles and Practice of Infectious Diseases. 9 ed. Philadelphia: Churchill Livingtone Elsevier; 2019 p. 2865–92.

[3] Salazar JC, Hazlett KR, Radolf JD. The immune response to infection with *Treponema pallidum*, the stealth pathogen. Microbes Infect. 2002;4(11):1133–40. 10.1016/s1286-4579(02)01638-6 12361913

[4] Radolf JD, Deka RK, Anand A, Smajs D, Norgard MV, Yang XF. *Treponema pallidum*, the syphilis spirochete: making a living as a stealth pathogen. Nat Rev Microbiol. 2016. 10.1038/nrmicro.2016.141 PMC5106329 27721440

[5] Workowski KA, Bolan GA, Centers for Disease C, Prevention. Sexually transmitted diseases treatment guidelines, 2015. MMWR Recomm Rep. 2015;64(RR-03):1–137. PMC5885289 26042815

[6] Patton ME, Su JR, Nelson R, Weinstock H, Centers for Disease C, Prevention. Primary and secondary syphilis—United States, 2005–2013. MMWR Morb Mortal Wkly Rep. 2014;63(18):402–6. 24807239 PMC5779405

[7] Peeling RW, Mabey D, Kamb ML, Chen XS, Radolf JD, Benzaken AS. Syphilis. Nat Rev Dis Primers. 2017;3:17073 10.1038/nrdp.2017.73 29022569 PMC5809176

[8] Gottlieb SL, Deal CD, Giersing B, Rees H, Bolan G, Johnston C, et al The global roadmap for advancing development of vaccines against sexually transmitted infections: Update and next steps. Vaccine. 2016;34(26):2939–47. 10.1016/j.vaccine.2016.03.111 27105564 PMC6759054

[9] Lithgow KV, Cameron CE. Vaccine development for syphilis. Expert Rev Vaccines. 2017;16(1):37–44. 10.1080/14760584.2016.1203262 27328030 PMC5513191

[10] Norris SJ, Cox DL, Weinstock GM. Biology of *Treponema pallidum*: correlation of functional activities with genome sequence data. J Mol Microbiol Biotechnol. 2001;3(1):37–62. 11200228

[11] Radolf JD, Hazlett KRO, Lukehart SA. Pathogenesis of Syphilis. In: Radolf JD, Lukehart SA, editors. Pathogenic Treponemes: Cellular and Molecular Biology. Norfolk,UK: Caister Academic Press; 2006 p. 197–236.

[12] Lukehart SA. Scientific monogamy: thirty years dancing with the same bug: 2007 Thomas Parran Award Lecture. Sex Transm Dis. 2008;35(1):2–7. 10.1097/OLQ.0b013e318162c4f2 18157060

[13] Cruz AR, Ramirez LG, Zuluaga AV, Pillay A, Abreu C, Valencia CA, et al Immune evasion and recognition of the syphilis spirochete in blood and skin of secondary syphilis patients: two immunologically distinct compartments. PLoS Negl Trop Dis. 2012;6(7):e1717 10.1371/journal.pntd.0001717 22816000 PMC3398964

[14] Hawley KL, Cruz AR, Benjamin SJ, La Vake CJ, Cervantes JL, LeDoyt M, et al IFN-g enhances CD64-potentiated phagocytosis of *Treponema pallidum* opsonized with human syphilitic serum by human macrophages. Front Immunol. 2017;8:1227 10.3389/fimmu.2017.01227 29051759 PMC5633599

[15] Marra CM, Tantalo LC, Sahi SK, Dunaway SB, Lukehart SA. Reduced *Treponema pallidum*-specific opsonic antibody activity in HIV-infected patients with syphilis. J Infect Dis. 2016;213(8):1348–54. 10.1093/infdis/jiv591 26655298 PMC4799670

[16] Radolf JD, Kumar S. The T*reponema pallidum* outer membrane. Curr Top Microbiol Immunol. 2018;415:1–38. 10.1007/82_2017_44 28849315 PMC5924592

[17] Izard J, Renken C, Hsieh CE, Desrosiers DC, Dunham-Ems S, La Vake C, et al Cryo-electron tomography elucidates the molecular architecture of *Treponema pallidum*, the syphilis spirochete. J Bacteriol. 2009;191(24):7566–80. 10.1128/JB.01031-09 19820083 PMC2786590

[18] Liu J, Howell JK, Bradley SD, Zheng Y, Zhou ZH, Norris SJ. Cellular architecture of *Treponema pallidum*: novel flagellum, periplasmic cone, and cell envelope as revealed by cryo electron tomography. J Mol Biol. 2010;403(4):546–61. 10.1016/j.jmb.2010.09.020 20850455 PMC2957517

[19] Fraser CM, Norris SJ, Weinstock GM, White O, Sutton GG, Dodson R, et al Complete genome sequence of *Treponema pallidum*, the syphilis spirochete. Science. 1998;281(5375):375–88. 10.1126/science.281.5375.375 9665876

[20] Cameron CE. The *T*. *pallidum* outer membrane and outer membrane proteins. In: Radolf JD, Lukehart SA, editors. Pathogenic *Treponema*: Molecular and Cellular Biology Norwich,UK: Caister Academic Press; 2006 p. 237–66.

[21] Chamberlain NR, Brandt ME, Erwin AL, Radolf JD, Norgard MV. Major integral membrane protein immunogens of *Treponema pallidum* are proteolipids. Infect Immun. 1989;57(9):2872–7. 10.1128/IAI.57.9.2872-2877.1989 2668191 PMC313540

[22] Setubal JC, Reis M, Matsunaga J, Haake DA. Lipoprotein computational prediction in spirochaetal genomes. Microbiology. 2006;152(Pt 1):113–21. 10.1099/mic.0.28317-0 16385121 PMC2667199

[23] Schouls LM, Mout R, Dekker J, van Embden JD. Characterization of lipid-modified immunogenic proteins of *Treponema pallidum* expressed in *Escherichia coli*. Microb Pathog. 1989;7(3):175–88. 10.1016/0882-4010(89)90053-3 2693885

[24] Brinkman MB, McKevitt M, McLoughlin M, Perez C, Howell J, Weinstock GM, et al Reactivity of antibodies from syphilis patients to a protein array representing the *Treponema pallidum* proteome. J Clin Microbiol. 2006;44(3):888–91. 10.1128/JCM.44.3.888-891.2006 16517872 PMC1393150

[25] Chamberlain NR, DeOgny L, Slaughter C, Radolf JD, Norgard MV. Acylation of the 47-kilodalton major membrane immunogen of *Treponema pallidum* determines its hydrophobicity. Infect Immun. 1989;57(9):2878–85. 10.1128/IAI.57.9.2878-2885.1989 2668192 PMC313541

[26] Weigel LM, Radolf JD, Norgard MV. The 47-kDa major lipoprotein immunogen of *Treponema pallidum* is a penicillin-binding protein with carboxypeptidase activity. Proc Natl Acad Sci U S A. 1994;91(24):11611–5. 10.1073/pnas.91.24.11611 7972112 PMC45281

[27] Deka RK, Machius M, Norgard MV, Tomchick DR. Crystal structure of the 47-kDa lipoprotein of *Treponema pallidum* reveals a novel penicillin-binding protein. J Biol Chem. 2002;277(44):41857–64. 10.1074/jbc.M207402200 12196546

[28] Deka RK, Goldberg MS, Hagman KE, Norgard MV. The Tp38 (TpMglB-2) lipoprotein binds glucose in a manner consistent with receptor function in *Treponema pallidum*. J Bacteriol. 2004;186(8):2303–8. 10.1128/jb.186.8.2303-2308.2004 15060032 PMC412163

[29] Deka RK, Brautigam CA, Yang XF, Blevins JS, Machius M, Tomchick DR, et al The PnrA (Tp0319; TmpC) lipoprotein represents a new family of bacterial purine nucleoside receptor encoded within an ATP-binding cassette (ABC)-like operon in *Treponema pallidum*. J Biol Chem. 2006;281(12):8072–81. 10.1074/jbc.M511405200 16418175

[30] Desrosiers DC, Sun YC, Zaidi AA, Eggers CH, Cox DL, Radolf JD. The general transition metal (Tro) and Zn2+ (Znu) transporters in *Treponema pallidum*: analysis of metal specificities and expression profiles. Mol Microbiol. 2007;65(1):137–52. 10.1111/j.1365-2958.2007.05771.x 17581125

[31] Machius M, Brautigam CA, Tomchick DR, Ward P, Otwinowski Z, Blevins JS, et al Structural and biochemical basis for polyamine binding to the Tp0655 lipoprotein of *Treponema pallidum*: putative role for Tp0655 (TpPotD) as a polyamine receptor. J Mol Biol. 2007;373(3):681–94. 10.1016/j.jmb.2007.08.018 17868688 PMC2094014

[32] Brautigam CA, Deka RK, Liu WZ, Norgard MV. The Tp0684 (MglB-2) lipoprotein of *Treponema pallidum*: a glucose-binding protein with divergent topology. PLoS One. 2016;11(8):e0161022 10.1371/journal.pone.0161022 27536942 PMC4990184

[33] Brinkman MB, McGill MA, Pettersson J, Rogers A, Matejkova P, Smajs D, et al A novel *Treponema pallidum* antigen, TP0136, is an outer membrane protein that binds human fibronectin. Infect Immun. 2008;76(5):1848–57. 10.1128/IAI.01424-07 18332212 PMC2346692

[34] Ke W, Molini BJ, Lukehart SA, Giacani L. *Treponema pallidum* subsp. *pallidum* TP0136 protein is heterogeneous among isolates and binds cellular and plasma fibronectin via its NH2-terminal end. PLoS Negl Trop Dis. 2015;9(3):e0003662 10.1371/journal.pntd.0003662 25793702 PMC4368718

[35] Chan K, Nasereddin T, Alter L, Centurion-Lara A, Giacani L, Parveen N. *Treponema pallidum* lipoprotein TP0435 expressed in Borrelia burgdorferi produces multiple surface/periplasmic isoforms and mediates adherence. Sci Rep. 2016;6:25593 10.1038/srep25593 27161310 PMC4861935

[36] Luthra A, Anand A, Radolf JD. *Treponema pallidum* in gel microdroplets: a method for topological analysis of BamA (TP0326) and localization of rare outer membrane proteins. Methods Mol Biol. 2015;1329:67–75. 10.1007/978-1-4939-2871-2_6 26427677

[37] Cox DL, Akins DR, Porcella SF, Norgard MV, Radolf JD. *Treponema pallidum* in gel microdroplets: a novel strategy for investigation of treponemal molecular architecture. Mol Microbiol. 1995;15(6):1151–64. 10.1111/j.1365-2958.1995.tb02288.x 7623668

[38] Cox DL, Luthra A, Dunham-Ems S, Desrosiers DC, Salazar JC, Caimano MJ, et al Surface immunolabeling and consensus computational framework to identify candidate rare outer membrane proteins of *Treponema pallidum*. Infect Immun. 2010;78(12):5178–94. 10.1128/IAI.00834-10 20876295 PMC2981305

[39] Brautigam CA, Deka RK, Liu WZ, Norgard MV. Insights into the potential function and membrane organization of the TP0435 (Tp17) lipoprotein from *Treponema pallidum* derived from structural and biophysical analyses. Protein Sci. 2015;24(1):11–9. 10.1002/pro.2576 25287511 PMC4282407

[40] Simpson BW, Trent MS. Emerging roles for NlpE as a sensor for lipoprotein maturation and transport to the outer membrane in *Escherichia coli*. mBio. 2019;10(3). 10.1128/mBio.01302-19 PMC6593411 31239385

[41] Cameron CE, Brouwer NL, Tisch LM, Kuroiwa JM. Defining the interaction of the *Treponema pallidum* adhesin Tp0751 with laminin. Infect Immun. 2005;73(11):7485–94. 10.1128/IAI.73.11.7485-7494.2005 16239550 PMC1273862

[42] Houston S, Hof R, Francescutti T, Hawkes A, Boulanger MJ, Cameron CE. Bifunctional role of the *Treponema pallidum* extracellular matrix binding adhesin Tp0751. Infect Immun. 2011;79(3):1386–98. 10.1128/IAI.01083-10 21149586 PMC3067502

[43] Houston S, Hof R, Honeyman L, Hassler J, Cameron CE. Activation and proteolytic activity of the *Treponema pallidum* metalloprotease, pallilysin. PLoS Pathog. 2012;8(7):e1002822 10.1371/journal.ppat.1002822 22910436 PMC3406077

[44] Houston S, Russell S, Hof R, Roberts AK, Cullen P, Irvine K, et al The multifunctional role of the pallilysin-associated *Treponema pallidum* protein, Tp0750, in promoting fibrinolysis and extracellular matrix component degradation. Mol Microbiol. 2014;91(3):618–34. 10.1111/mmi.12482 24303899 PMC3954913

[45] Parker ML, Houston S, Petrosova H, Lithgow KV, Hof R, Wetherell C, et al The structure of *Treponema pallidum* Tp0751 (pallilysin) reveals a non-canonical lipocalin fold that mediates adhesion to extracellular matrix components and interactions with host cells. PLoS Pathog. 2016;12(9):e1005919 10.1371/journal.ppat.1005919 27683203 PMC5040251

[46] Kao WA, Petrosova H, Ebady R, Lithgow KV, Rojas P, Zhang Y, et al Identification of Tp0751 (Pallilysin) as a *Treponema pallidum* vascular adhesin by heterologous expression in the Lyme disease spirochete. Sci Rep. 2017;7(1):1538 10.1038/s41598-017-01589-4 28484210 PMC5431505

[47] Lithgow KV, Hof R, Wetherell C, Phillips D, Houston S, Cameron CE. A defined syphilis vaccine candidate inhibits dissemination of *Treponema pallidum* subspecies *pallidum*. Nat Commun. 2017;8:14273 10.1038/ncomms14273 28145405 PMC5296639

[48] Bishop RE. The bacterial lipocalins. Biochim Biophys Acta. 2000;1482(1–2):73–83. 10.1016/s0167-4838(00)00138-2 11058749

[49] Schiefner A, Skerra A. The menagerie of human lipocalins: a natural protein scaffold for molecular recognition of physiological compounds. Acc Chem Res. 2015;48(4):976–85. 10.1021/ar5003973 25756749

[50] David G, Blondeau K, Schiltz M, Penel S, Lewit-Bentley A. YodA from Escherichia coli is a metal-binding, lipocalin-like protein. J Biol Chem. 2003;278(44):43728–35. 10.1074/jbc.M304484200 12909634

[51] Handa N, Terada T, Doi-Katayama Y, Hirota H, Tame JR, Park SY, et al Crystal structure of a novel polyisoprenoid-binding protein from *Thermus thermophilus* HB8. Protein Sci. 2005;14(4):1004–10. 10.1110/ps.041183305 15741337 PMC2253440

[52] Donnarumma D, Golfieri G, Brier S, Castagnini M, Veggi D, Bottomley MJ, et al *Neisseria meningitis* GNA1030 is a ubiquinone-8 binding protein. FASEB J. 2015;29(6):2260–7. 10.1096/fj.14-263954 25713028

[53] Campanacci V, Bishop RE, Blangy S, Tegoni M, Cambillau C. The membrane bound bacterial lipocalin Blc is a functional dimer with binding preference for lysophospholipids. FEBS Lett. 2006;580(20):4877–83. 10.1016/j.febslet.2006.07.086 16920109 PMC5007124

[54] Li P, Rivera-Cancel G, Kinch LN, Salomon D, Tomchick DR, Grishin NV, et al Bile salt receptor complex activates a pathogenic type III secretion system. Elife. 2016;5. 10.7554/eLife.15718 PMC4933562 27377244

[55] El-Halfawy OM, Klett J, Ingram RJ, Loutet SA, Murphy ME, Martin-Santamaria S, et al Antibiotic capture by bacterial lipocalins uncovers an extracellular mechanism of intrinsic antibiotic resistance. MBio. 2017;8(2). 10.1128/mBio.00225-17 PMC5350466 28292982

[56] Kelley LA, Mezulis S, Yates CM, Wass MN, Sternberg MJ. The Phyre2 web portal for protein modeling, prediction and analysis. Nat Protoc. 2015;10(6):845–58. 10.1038/nprot.2015.053 25950237 PMC5298202

[57] Ishida T, Kinoshita K. PrDOS: prediction of disordered protein regions from amino acid sequence. Nucleic Acids Res. 2007;35(Web Server):W460–W4. 17567614 10.1093/nar/gkm363 PMC1933209

[58] Dosztányi Z. Prediction of protein disorder based on IUPred. Protein Science. 2018;27(1):331–40. 10.1002/pro.3334 29076577 PMC5734386

[59] Xue B, Dunbrack RL, Williams RW, Dunker AK, Uversky VN. PONDR-FIT: a meta-predictor of intrinsically disordered amino acids. Biochim Biophys Acta. 2010;1804(4):996–1010. 10.1016/j.bbapap.2010.01.011 20100603 PMC2882806

[60] Gorba C, Tama F. Normal mode flexible fitting of high-resolution structures of biological molecules toward SAXS data. Bioinform Biol Insights. 2010;4:43–54. 10.4137/bbi.s4551 20634984 PMC2901630

[61] Svergun DI. Restoring low resolution structure of biological macromolecules from solution scattering using simulated annealing. Biophys J. 1999;76(6):2879–86. 10.1016/S0006-3495(99)77443-6 10354416 PMC1300260

[62] Volkov VV, Svergun DI. Uniqueness of ab *initio* shape determination in small-angle scattering. J Appl Crystallog. 2003;36:860–4. 10.1107/S0021889809000338 PMC5023043 27630371

[63] Grzyb J, Latowski D, Strzalka K. Lipocalins—a family portrait. J Plant Physiol. 2006;163(9):895–915. 10.1016/j.jplph.2005.12.007 16504339

[64] Greene LH, Chrysina ED, Irons LI, Papageorgiou AC, Acharya KR, Brew K. Role of conserved residues in structure and stability: tryptophans of human serum retinol-binding protein, a model for the lipocalin superfamily. Protein Sci. 2001;10(11):2301–16. 10.1110/ps.22901 11604536 PMC2374051

[65] Flower DR, North AC, Sansom CE. The lipocalin protein family: structural and sequence overview. Biochim Biophys Acta. 2000;1482(1–2):9–24. 10.1016/s0167-4838(00)00148-5 11058743

[66] Munussami G, Sokalingam S, Sriramulu DK, Lee S-G. Identification of common and distinct features of ligand-binding sites in kernel and outlier lipocalins. Journal of Industrial and Engineering Chemistry. 2019;78:344–51.

[67] Lakshmi B, Mishra M, Srinivasan N, Archunan G. Structure-based phylogenetic analysis of the lipocalin superfamily. PLoS One. 2015;10(8):e0135507 10.1371/journal.pone.0135507 26263546 PMC4532494

[68] Ganfornina MD, Gutierrez G, Bastiani M, Sanchez D. A phylogenetic analysis of the lipocalin protein family. Mol Biol Evol. 2000;17(1):114–26. 10.1093/oxfordjournals.molbev.a026224 10666711

[69] Balaji S, Srinivasan N. Comparison of sequence-based and structure-based phylogenetic trees of homologous proteins: Inferences on protein evolution. J Biosci. 2007;32(1):83–96. 10.1007/s12038-007-0008-1 17426382

[70] Agarwal G, Rajavel M, Gopal B, Srinivasan N. Structure-based phylogeny as a diagnostic for functional characterization of proteins with a cupin fold. PLoS One. 2009;4(5):e5736 10.1371/journal.pone.0005736 19478949 PMC2684688

[71] Holm L, Rosenstrom P. Dali server: conservation mapping in 3D. Nucleic Acids Res. 2010;38(Web Server issue):W545–9. 10.1093/nar/gkq366 20457744 PMC2896194

[72] Hirano Y, Hossain MM, Takeda K, Tokuda H, Miki K. Structural studies of the Cpx pathway activator NlpE on the outer membrane of *Escherichia coli*. Structure. 2007;15(8):963–76. 10.1016/j.str.2007.06.014 17698001

[73] Wu Y, Punta M, Xiao R, Acton TB, Sathyamoorthy B, Dey F, et al NMR structure of lipoprotein YxeF from *Bacillus subtilis* reveals a calycin fold and distant homology with the lipocalin Blc from *Escherichia coli*. PLoS One. 2012;7(6):e37404 10.1371/journal.pone.0037404 22693626 PMC3367933

[74] Lithgow KV, Church B, Gomez A, Tsao E, Houston S, Swayne LA, et al Identification of the neuroinvasive pathogen host target, LamR, as an endothelial receptor for the *Treponema pallidum* adhesin Tp0751. mSphere. 2020;5(2). 10.1128/mSphere.00195-20 PMC7113585 32238570

[75] Bao GH, Ho CT, Barasch J. The Ligands of Neutrophil Gelatinase-Associated Lipocalin. RSC Adv. 2015;5(126):104363–74. 10.1039/C5RA18736B 27617081 PMC5014392

[76] Goetz DH, Holmes MA, Borregaard N, Bluhm ME, Raymond KN, Strong RK. The neutrophil lipocalin NGAL is a bacteriostatic agent that interferes with siderophore-mediated iron acquisition. Mol Cell. 2002;10(5):1033–43. 10.1016/s1097-2765(02)00708-6 12453412

[77] Yang J, Roy A, Zhang Y. Protein-ligand binding site recognition using complementary binding-specific substructure comparison and sequence profile alignment. Bioinformatics. 2013;29(20):2588–95. 10.1093/bioinformatics/btt447 23975762 PMC3789548

[78] Yang J, Roy A, Zhang Y. BioLiP: a semi-manually curated database for biologically relevant ligand-protein interactions. Nucleic Acids Res. 2013;41(Database issue):D1096–103. 10.1093/nar/gks966 23087378 PMC3531193

[79] Wass MN, Kelley LA, Sternberg MJ. 3DLigandSite: predicting ligand-binding sites using similar structures. Nucleic Acids Res. 2010;38(Web Server issue):W469–73. 10.1093/nar/gkq406 20513649 PMC2896164

[80] Tian W, Chen C, Lei X, Zhao J, Liang J. CASTp 3.0: computed atlas of surface topography of proteins. Nucleic Acids Res. 2018;46(W1):W363–W7. 10.1093/nar/gky473 29860391 PMC6031066

[81] Morris GM, Huey R, Lindstrom W, Sanner MF, Belew RK, Goodsell DS, et al AutoDock4 and AutoDockTools4: Automated docking with selective receptor flexibility. J Comput Chem. 2009;30(16):2785–91. 10.1002/jcc.21256 19399780 PMC2760638

[82] Matthews HM, Yang TK, Jenkin HM. Unique lipid composition of *Treponema pallidum* (Nichols virulent strain). Infect Immun. 1979;24(3):713–9. 10.1128/IAI.24.3.713-719.1979 381199 PMC414365

[83] Belisle JT, Brandt ME, Radolf JD, Norgard MV. Fatty acids of *Treponema pallidum* and *Borrelia burgdorferi* lipoproteins. J Bacteriol. 1994;176(8):2151–7. 10.1128/jb.176.8.2151-2157.1994 8157583 PMC205333

[84] Laskowski RA, Swindells MB. LigPlot+: multiple ligand-protein interaction diagrams for drug discovery. J Chem Inf Model. 2011;51(10):2778–86. 10.1021/ci200227u 21919503

[85] Shen J, Sheng X, Chang Z, Wu Q, Wang S, Xuan Z, et al Iron metabolism regulates p53 signaling through direct heme-p53 interaction and modulation of p53 localization, stability, and function. Cell Rep. 2014;7(1):180–93. 10.1016/j.celrep.2014.02.042 24685134 PMC4219651

[86] Osbak KK, Houston S, Lithgow KV, Meehan CJ, Strouhal M, Smajs D, et al Characterizing the syphilis-causing *Treponema pallidum* ssp. *pallidum* proteome using complementary mass spectrometry. PLoS Negl Trop Dis. 2016;10(9):e0004988 10.1371/journal.pntd.0004988 27606673 PMC5015957

[87] Radolf JD, Chamberlain NR, Clausell A, Norgard MV. Identification and localization of integral membrane proteins of virulent *Treponema pallidum* subsp. *pallidum* by phase partitioning with the nonionic detergent triton X-114. Infect Immun. 1988;56(2):490–8. 10.1128/IAI.56.2.490-498.1988 3276627 PMC259309

[88] Luthra A, Zhu G, Desrosiers DC, Eggers CH, Mulay V, Anand A, et al The transition from closed to open conformation of *Treponema pallidum* outer membrane-associated lipoprotein TP0453 involves membrane sensing and integration by two amphipathic helices. J Biol Chem. 2011;286(48):41656–68. 10.1074/jbc.M111.305284 21965687 PMC3308875

[89] Hazlett KR, Cox DL, Decaffmeyer M, Bennett MP, Desrosiers DC, La Vake CJ, et al TP0453, a concealed outer membrane protein of *Treponema pallidum*, enhances membrane permeability. J Bacteriol. 2005;187(18):6499–508. 10.1128/JB.187.18.6499-6508.2005 16159783 PMC1236642

[90] Radolf JD, Lukehart SA. Immunology of Syphilis. In: Radolf JD, Lukehart SA, editors. Pathogenic Treponemes: Cellular and Molecular Biology. Norfolk, UK: Caister Academic Press; 2006 p. 285–322.

[91] Sell S, Norris SJ. The biology, pathology, and immunology of syphilis. Int Rev Exp Pathol. 1983;24:203–76. 6840996

[92] Alderete JF, Baseman JB. Surface-associated host proteins on virulent *Treponema pallidum*. Infect Immun. 1979;26(3):1048–56. 10.1128/IAI.26.3.1048-1056.1979 93574 PMC414726

[93] Kumar S, Caimano MJ, Anand A, Dey A, Hawley K, Ledoyt ME, et al Sequence variation of rare outer membrane protein β-barrel domains in clinical strains provides insights into the evolution of Treponema pallidum subsp. *pallidum*, the syphilis spirochete. MBio. 2018;9(3):e01006–18. 10.1128/mBio.01006-18 29895642 PMC6016234

[94] Anand A, LeDoyt M, Karanian C, Luthra A, Koszelak-Rosenblum M, Malkowski MG, et al Bipartite topology of *Treponema pallidum* repeat proteins C/D and I: outer membrane insertion, trimerization, and porin function require a C-terminal β-barrel domain. J Biol Chem. 2015;290(19):12313–31. 10.1074/jbc.M114.629188 25805501 PMC4424362

[95] Luthra A, Anand A, Hawley KL, LeDoyt M, La Vake CJ, Caimano MJ, et al A homology model reveals novel structural features and an immunodominant surface loop/opsonic target in the *Treponema pallidum* BamA ortholog TP_0326. J Bacteriol. 2015;197(11):1906–20. 10.1128/JB.00086-15 25825429 PMC4420902

[96] Radolf JD, Norgard MV. Pathogen specificity of *Treponema pallidum* subsp. *pallidum* integral membrane proteins identified by phase partitioning with Triton X-114. Infect Immun. 1988;56(7):1825–8. 10.1128/IAI.56.7.1825-1828.1988 3290110 PMC259484

[97] Deka RK, Brautigam CA, Tomson FL, Lumpkins SB, Tomchick DR, Machius M, et al Crystal structure of the Tp34 (TP0971) lipoprotein of *Treponema pallidum*: implications of its metal-bound state and affinity for human lactoferrin. J Biol Chem. 2007;282(8):5944–58. 10.1074/jbc.M610215200 17192261

[98] Komarudin AG, Driessen AJM. SecA-mediated protein translocation through the SecYEG Channel. Microbiol Spectr. 2019;7(4). 10.1128/microbiolspec.psib-0028-2019 PMC10957188 31373268

[99] Hooda Y, Lai CC, Judd A, Buckwalter CM, Shin HE, Gray-Owen SD, et al Slam is an outer membrane protein that is required for the surface display of lipidated virulence factors in *Neisseria*. Nat Microbiol. 2016;1:16009 10.1038/nmicrobiol.2016.9 27572441

[100] Hooda Y, Lai CCL, Moraes TF. Identification of a large family of Slam-dependent surface lipoproteins in Gram-negative bacteria. Front Cell Infect Microbiol. 2017;7:207 10.3389/fcimb.2017.00207 28620585 PMC5449769

[101] Radolf JD, Caimano MJ, Stevenson B, Hu LT. Of ticks, mice and men: understanding the dual-host lifestyle of Lyme disease spirochaetes. Nat Rev Microbiol. 2012;10(2):87–99. 10.1038/nrmicro2714 22230951 PMC3313462

[102] Dowdell AS, Murphy MD, Azodi C, Swanson SK, Florens L, Chen S, et al Comprehensive spatial analysis of the *Borrelia burgdorferi* lipoproteome reveals a compartmentalization bias toward the bacterial surface. J Bacteriol. 2017;199(6). 10.1128/JB.00658-16 PMC5331670 28069820

[103] Zuckert WR. Secretion of bacterial lipoproteins: through the cytoplasmic membrane, the periplasm and beyond. Biochim Biophys Acta. 2014;1843(8):1509–16. 10.1016/j.bbamcr.2014.04.022 24780125 PMC4070597

[104] Cantini F, Veggi D, Dragonetti S, Savino S, Scarselli M, Romagnoli G, et al Solution structure of the factor H-binding protein, a survival factor and protective antigen of Neisseria meningitidis. J Biol Chem. 2009;284(14):9022–6. 10.1074/jbc.C800214200 19196709 PMC2666550

[105] Sia AK, Allred BE, Raymond KN. Siderocalins: Siderophore binding proteins evolved for primary pathogen host defense. Curr Opin Chem Biol. 2013;17(2):150–7. 10.1016/j.cbpa.2012.11.014 23265976 PMC3634885

[106] Karnaukhova E, Rutardottir S, Rajabi M, Wester Rosenlof L, Alayash AI, Akerstrom B. Characterization of heme binding to recombinant alpha1-microglobulin. Front Physiol. 2014;5:465 10.3389/fphys.2014.00465 25538624 PMC4255499

[107] Choby JE, Skaar EP. Heme synthesis and acquisition in bacterial pathogens. J Mol Biol. 2016;428(17):3408–28. 10.1016/j.jmb.2016.03.018 27019298 PMC5125930

[108] Pi H, Helmann JD. Ferrous iron efflux systems in bacteria. Metallomics. 2017;9(7):840–51. 10.1039/c7mt00112f 28604884 PMC5675029

[109] Dyson HJ, Wright PE. Intrinsically unstructured proteins and their functions. Nat Rev Mol Cell Biol. 2005;6(3):197–208. 10.1038/nrm1589 15738986

[110] Yang J, Gao M, Xiong J, Su Z, Huang Y. Features of molecular recognition of intrinsically disordered proteins via coupled folding and binding. Protein Sci. 2019;28(11):1952–65. 10.1002/pro.3718 31441158 PMC6798136

[111] Pappu RV. Cell signaling, division, and organization mediated by intrinsically disordered proteins. Seminars in Cell & Developmental Biology. 2015;37:1–2. 25703796 10.1016/j.semcdb.2015.01.003

[112] Lobanov MY, Galzitskaya OV. How common Is disorder? Occurrence of disordered residues in four domains of life. Int J Mol Sci. 2015;16(8):19490–507. 10.3390/ijms160819490 26295225 PMC4581309

[113] Loftus SR, Walker D, Mate MJ, Bonsor DA, James R, Moore GR, et al Competitive recruitment of the periplasmic translocation portal TolB by a natively disordered domain of colicin E9. Proc Natl Acad Sci U S A. 2006;103(33):12353–8. 10.1073/pnas.0603433103 16894158 PMC1567883

[114] Asiani KR, Williams H, Bird L, Jenner M, Searle MS, Hobman JL, et al SilE is an intrinsically disordered periplasmic "molecular sponge" involved in bacterial silver resistance. Mol Microbiol. 2016;101(5):731–42. 10.1111/mmi.13399 27085056 PMC5008109

[115] Housden NG, Rassam P, Lee S, Samsudin F, Kaminska R, Sharp C, et al Directional porin binding of intrinsically disordered protein sequences promotes colicin epitope display in the bacterial periplasm. Biochemistry. 2018;57(29):4374–81. 10.1021/acs.biochem.8b00621 29949342 PMC6093495

[116] Kopp DR, Postle K. The Intrinsically disordered region of ExbD is required for signal transduction. Journal of Bacteriology. 2020. 10.1128/JB.00687-19 PMC7167468 31932309

[117] Shin WH, Kihara D. 55 Years of the Rossmann fold. Methods Mol Biol. 2019;1958:1–13. 10.1007/978-1-4939-9161-7_1 30945211

[118] Cruz AR, Pillay A, Zuluaga AV, Ramirez LG, Duque JE, Aristizabal GE, et al Secondary syphilis in Cali, Colombia: new concepts in disease pathogenesis. PLoS Negl Trop Dis. 2010;4(5):e690 10.1371/journal.pntd.0000690 20502522 PMC2872645

[119] Spiliotis M. Inverse fusion PCR cloning. PLoS One. 2012;7(4):e35407 10.1371/journal.pone.0035407 22530019 PMC3328455

[120] Allaire M, Yang L. Biomolecular solution X-ray scattering at the National Synchrotron Light Source. J Synchrotron Radiat. 2011;18(1):41–4. 10.1107/S0909049510036022 21169689 PMC3004252

[121] Konarev PV, Volkov VV, Sokolova AV, Koch MH, Svergun DI. Primus: A Windows PC-based system for small-angle scattering data analysis. J Appl Crystallog. 2003;36:1277–82.

[122] Svergun DI. Determination of the regularization parameter in indirect-transform methods using perceptual criteria. J Appl Crystallog. 1992;25:495–503.

[123] Schneidman-Duhovny D, Hammel M, Tainer JA, Sali A. FoXS, FoXSDock and MultiFoXS: Single-state and multi-state structural modeling of proteins and their complexes based on SAXS profiles. Nucleic Acids Res. 2016;44(W1):W424–9. 10.1093/nar/gkw389 27151198 PMC4987932

[124] Panjkovich A, Svergun DI. Deciphering conformational transitions of proteins by small angle X-ray scattering and normal mode analysis. Phys Chem Chem Phys. 2016;18(8):5707–19. 10.1039/c5cp04540a 26611321

[125] Petoukhov MV, Franke D, Shkumatov AV, Tria G, Kikhney AG, Gajda M, et al New developments in the ATSAS program package for small-angle scattering data analysis. J Appl Crystallogr. 2012;45(Pt 2):342–50. 10.1107/S0021889812007662 25484842 PMC4233345

[126] Emsley P, Lohkamp B, Scott WG, Cowtan K. Features and development of Coot. Acta Crystallographica Section D Biological Crystallography. 2010;66(4):486–501. 20383002 10.1107/S0907444910007493 PMC2852313

[127] Laskowski RA, Jablonska J, Pravda L, Varekova RS, Thornton JM. PDBsum: Structural summaries of PDB entries. Protein Sci. 2018;27(1):129–34. 10.1002/pro.3289 28875543 PMC5734310

[128] Janson G, Zhang C, Prado MG, Paiardini A. PyMod 2.0: improvements in protein sequence-structure analysis and homology modeling within PyMOL. Bioinformatics. 2017;33(3):444–6. 10.1093/bioinformatics/btw638 28158668

[129] Pei J, Kim BH, Grishin NV. PROMALS3D: a tool for multiple protein sequence and structure alignments. Nucleic Acids Res. 2008;36(7):2295–300. 10.1093/nar/gkn072 18287115 PMC2367709

[130] Robert X, Gouet P. Deciphering key features in protein structures with the new ENDscript server. Nucleic Acids Res. 2014;42(Web Server issue):W320–4. 10.1093/nar/gku316 24753421 PMC4086106

[131] Obradovic Z, Peng K, Vucetic S, Radivojac P, Brown CJ, Dunker AK. Predicting intrinsic disorder from amino acid sequence. Proteins. 2003;53 Suppl 6:566–72. 14579347 10.1002/prot.10532

[132] Peherstorfer S, Brewitz HH, Paul George AA, Wissbrock A, Adam JM, Schmitt L, et al Insights into mechanism and functional consequences of heme binding to hemolysin-activating lysine acyltransferase HlyC from Escherichia coli. Biochim Biophys Acta Gen Subj. 2018;1862(9):1964–72. 10.1016/j.bbagen.2018.06.012 29908817

[133] Desrosiers DC, Anand A, Luthra A, Dunham-Ems SM, LeDoyt M, Cummings MA, et al TP0326, a *Treponema pallidum* β-barrel assembly machinery A (BamA) orthologue and rare outer membrane protein. Mol Microbiol. 2011;80(6):1496–515. 10.1111/j.1365-2958.2011.07662.x 21488980 PMC3115443

[134] Anand A, Luthra A, Dunham-Ems S, Caimano MJ, Karanian C, LeDoyt M, et al TprC/D (Tp0117/131), a trimeric, pore-forming rare outer membrane protein of *Treponema pallidum*, has a bipartite domain structure. J Bacteriol. 2012;194(9):2321–33. 10.1128/JB.00101-12 22389487 PMC3347077

[135] Lukehart SA, Miller JN. Demonstration of the *in vitro* phagocytosis of *Treponema pallidum* by rabbit peritoneal macrophages. J Immunol. 1978;121(5):2014–24. 361893

[136] Hazlett KR, Sellati TJ, Nguyen TT, Cox DL, Clawson ML, Caimano MJ, et al The TprK protein of *Treponema pallidum* is periplasmic and is not a target of opsonic antibody or protective immunity. J Exp Med. 2001;193(9):1015–26. 10.1084/jem.193.9.1015 11342586 PMC2193430

[137] Sievers F, Wilm A, Dineen D, Gibson TJ, Karplus K, Li W, et al Fast, scalable generation of high-quality protein multiple sequence alignments using Clustal Omega. Mol Syst Biol. 2011;7:539 10.1038/msb.2011.75 21988835 PMC3261699

[138] Garcia-Boronat M, Diez-Rivero CM, Reinherz EL, Reche PA. PVS: a web server for protein sequence variability analysis tuned to facilitate conserved epitope discovery. Nucleic Acids Res. 2008;36(Web Server issue):W35–41. 10.1093/nar/gkn211 18442995 PMC2447719

[139] Lukehart SA, Marra CM. Isolation and laboratory maintenance of *Treponema pallidum*. Curr Protoc Microbiol. 2007;Chapter 12:Unit 12A 1. 10.1002/9780471729259.mc12a01s7 18770607

[140] Akins DR, Purcell BK, Mitra MM, Norgard MV, Radolf JD. Lipid modification of the 17-kilodalton membrane immunogen of *Treponema pallidum* determines macrophage activation as well as amphiphilicity. Infect Immun. 1993;61(4):1202–10. 10.1128/IAI.61.4.1202-1210.1993 8454324 PMC281349

[141] Chamberlain NR, Radolf JD, Hsu PL, Sell S, Norgard MV. Genetic and physicochemical characterization of the recombinant DNA-derived 47-kilodalton surface immunogen of *Treponema pallidum* subsp. *pallidum*. Infect Immun. 1988;56(1):71–8. 10.1128/IAI.56.1.71-78.1988 3275588 PMC259236

