# Supplementary figures and images for: Evidence that immunization with TP0751, a bipartite Treponema pallidum lipoprotein with an intrinsically disordered region and lipocalin fold, fails to protect in the rabbit model of experimental syphilis
Source: PLoS Pathog. 2020 Sep 16;16(9):e1008871. doi: 10.1371/journal.ppat.1008871 (PMC7521688; doi:10.1371/journal.ppat.1008871)

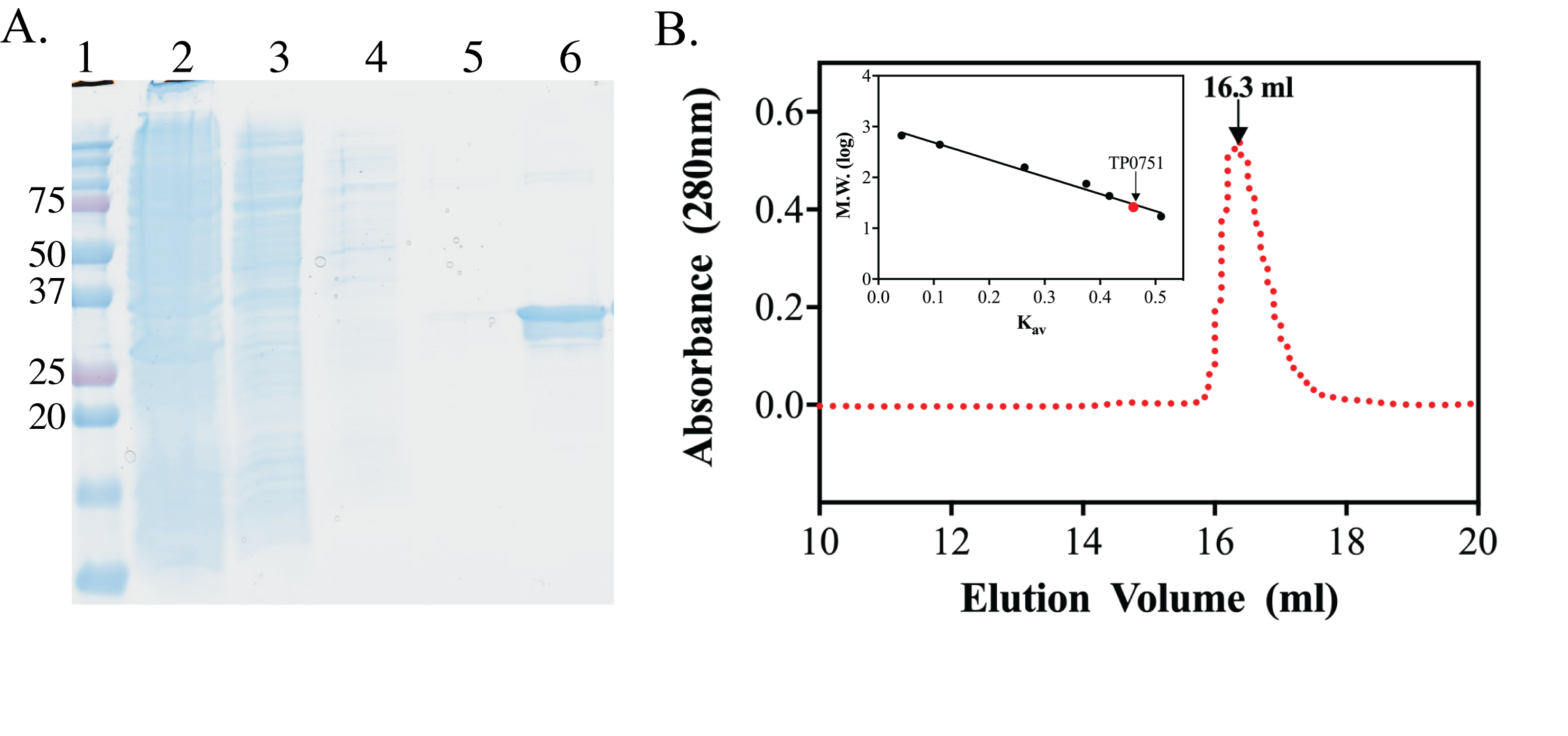

Supplement: S1 Fig — (A) Purification of recombinant TP075125-237 from E. coli under non-denaturing conditions using affinity chromatography. Lanes 1–6 represent molecular weight markers, supernatant of induced culture lysate, flow-through, 30 mM imidazole wash, 80 mM imidazole wash, and protein eluted in 300 mM imidazole from the nickel–nitrilotriacetic column, respectively. (B) Size-exclusion chromatography (SEC) of TP075125-237 produces a single peak corresponding to a molecular weight of ~25 kDa. The inset shows the SEC calibration curve calculated by a linear fit of known molecular weight (M.W.) standards as a function of measured partition coefficients (Kav). The red and black circles, respectively, show the partition coefficients of recombinant TP075125-237 and calibration standards. (TIF) [file ppat.1008871.s001.tif]

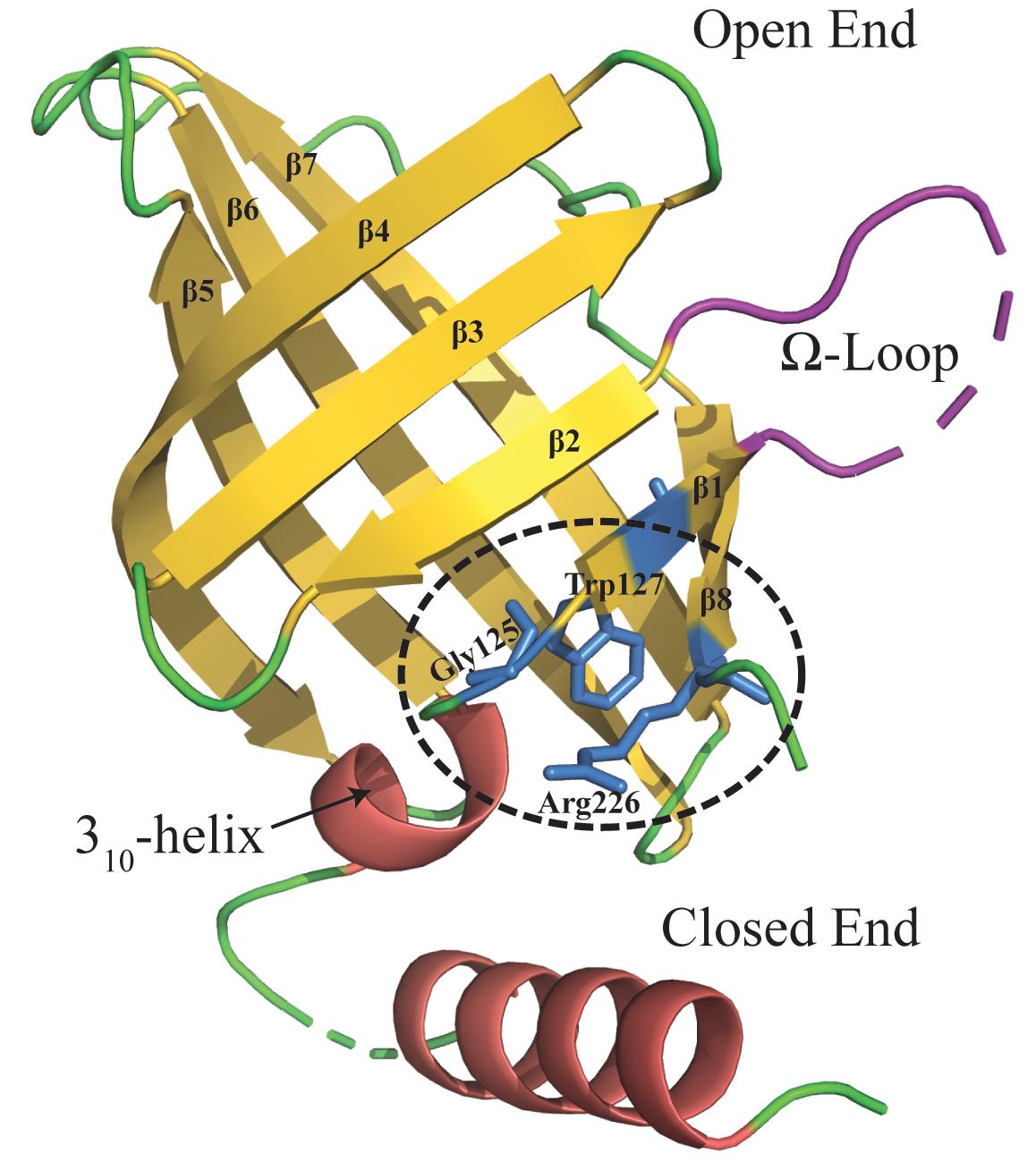

Supplement: S2 Fig — The lipocalin fold of TP075197-228 depicted as a ribbon model in which α helices are shown in red, β strands are shown in yellow, and loops are shown in green. The Ω-loop is shown in magenta. Residues of the calycin signature motif are outlined by the dashed oval and labeled. The arrow indicates a 310-helix located on the closed side of the β barrel. (TIF) [file ppat.1008871.s002.tif]

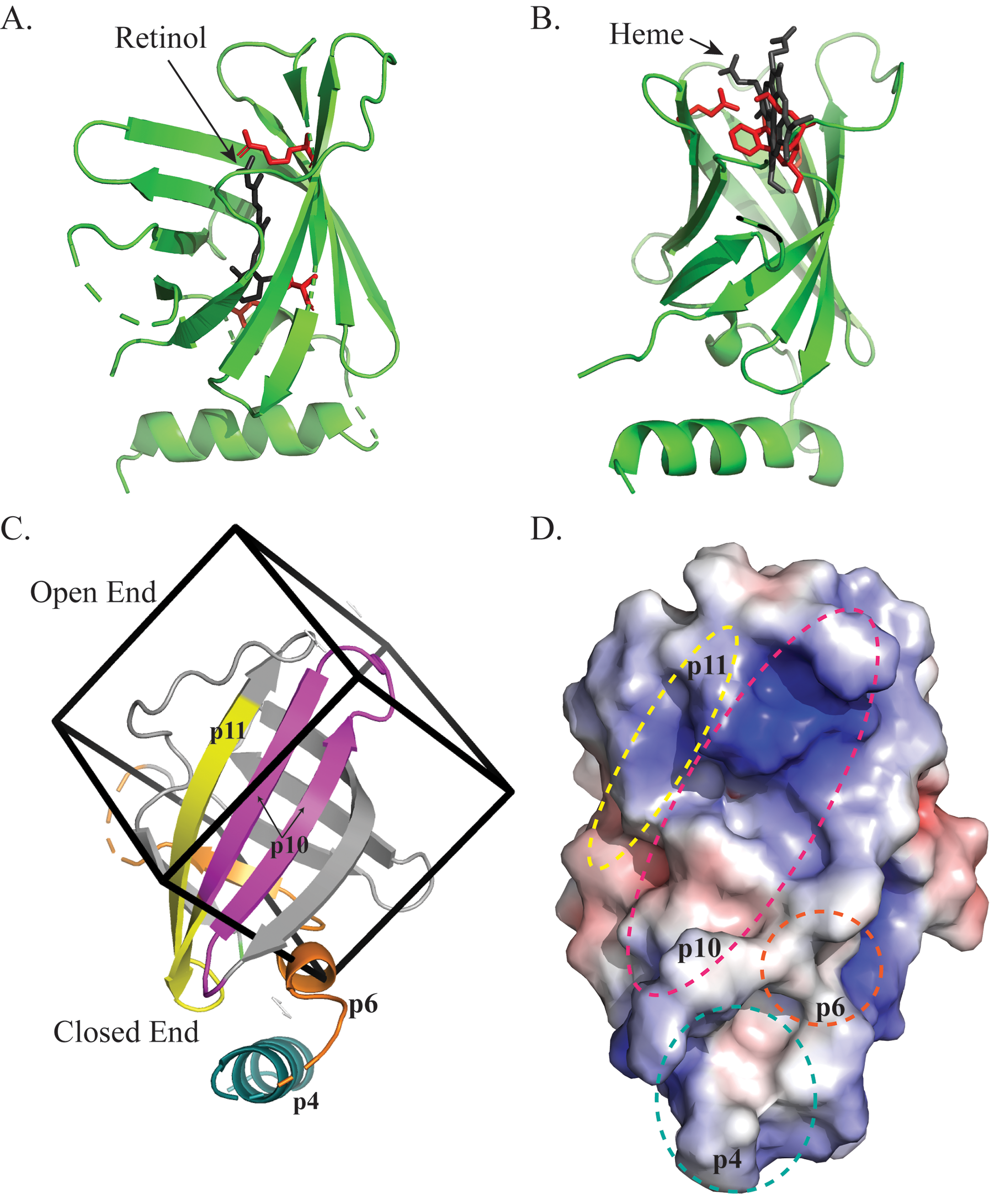

Supplement: S3 Fig — Ligand-binding site in TP075197-228 for (A) retinol and (B) heme predicted by COACH and 3DLigandSite. Residues of steric clashes are depicted as red sticks. (C) The AutoDock grid box (50 × 50 × 50 Å along the X, Y, and Z axes) used for docking calculations. The solid black box centered at coordinates X:72.769, Y:49.091, Z:34.004 (PDB ID: 5JK2) represents the coverage of the docking grid. Previously reported laminin-binding peptides [45] p4 (residues 97–111, cyan), p6 (residues 116–138, orange), p10 (residues 172–195, magenta) and p11 (residues 196–209, yellow) mapped onto the structure of TP075197-228. p10 also has also been reported to interact with LamR [74]. TP075197-228 is shown in a different orientation compared to other figures to facilitate presentation of the grid box and the locations of laminin-binding peptides. (D) Electrostatic potential of TP075197-228. Dashed lines indicate the location of different peptides on the surface of the TP075197-228 structure. Panels C and D are in the same orientation. (TIF) [file ppat.1008871.s003.tif]

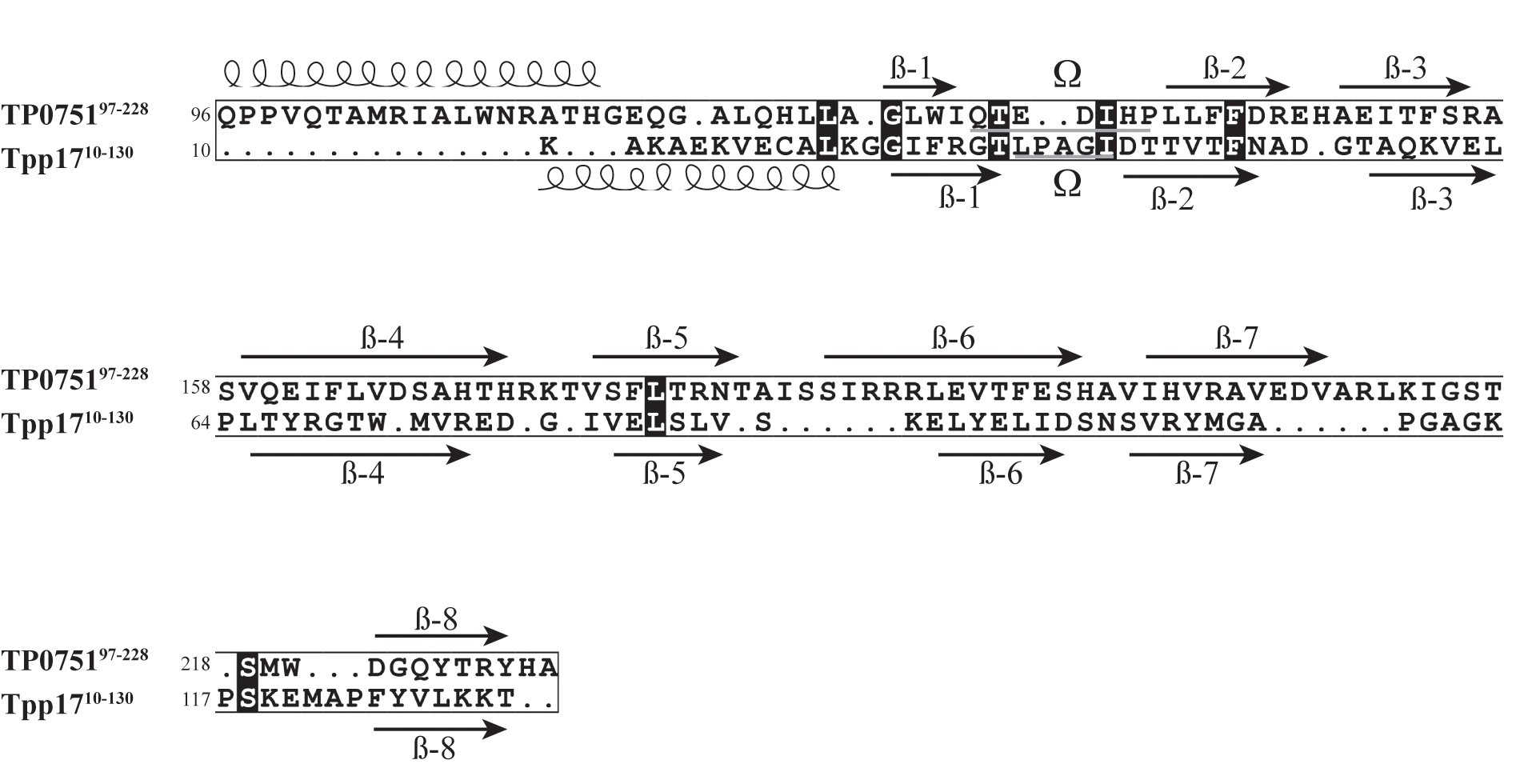

Supplement: S4 Fig — Identical residues are highlighted in black. The secondary structure elements (α-helix: spiral line; β strand: arrow) are shown for both proteins above and below their respective sequences. Residues of the Ω-type loop are underlined. (TIF) [file ppat.1008871.s004.tif]

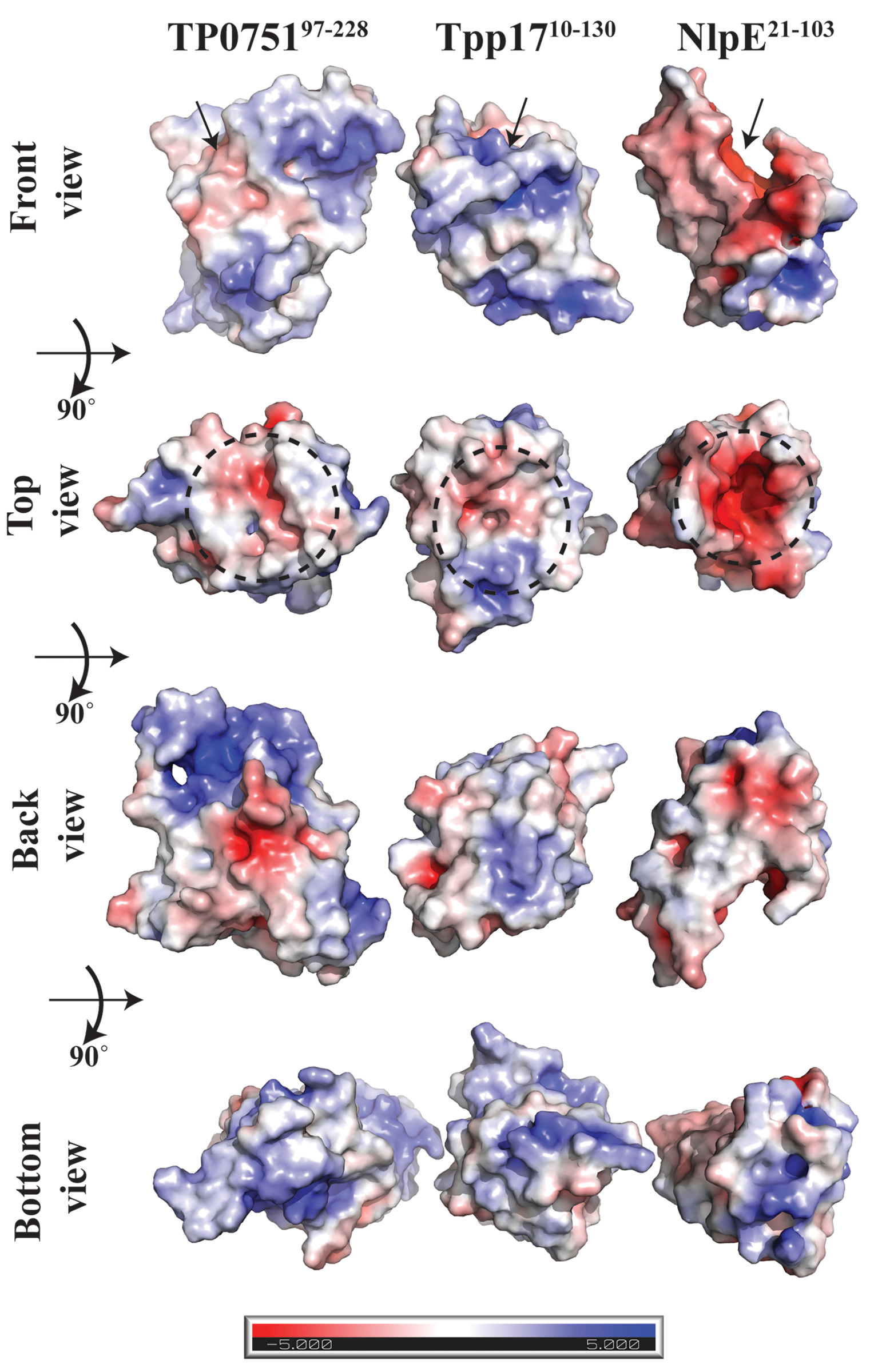

Supplement: S5 Fig — The surface is colored according to the local electrostatic potential (−5 kT to +5 kT), calculated using the ABPS plug in in PyMOL (https://pymol.org/). The open end of each β-barrel is indicated by an arrow in the front view. Dashed lines represent the exterior of barrel rim. (TIF) [file ppat.1008871.s005.tif]

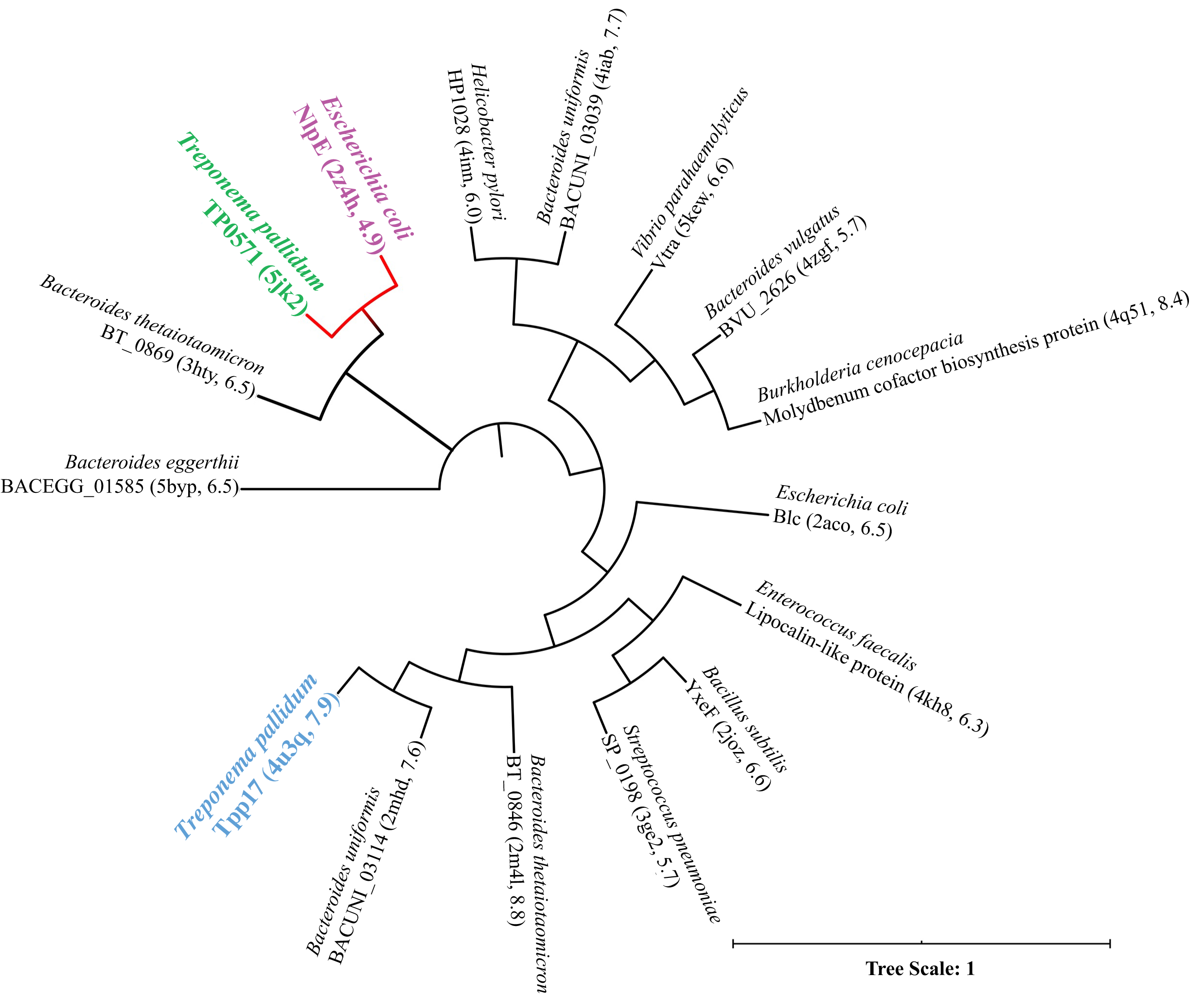

Supplement: S6 Fig — PDB IDs of bacterial lipocalins and Z-scores against TP075197-226 are in parentheses. (TIF) [file ppat.1008871.s006.tif]

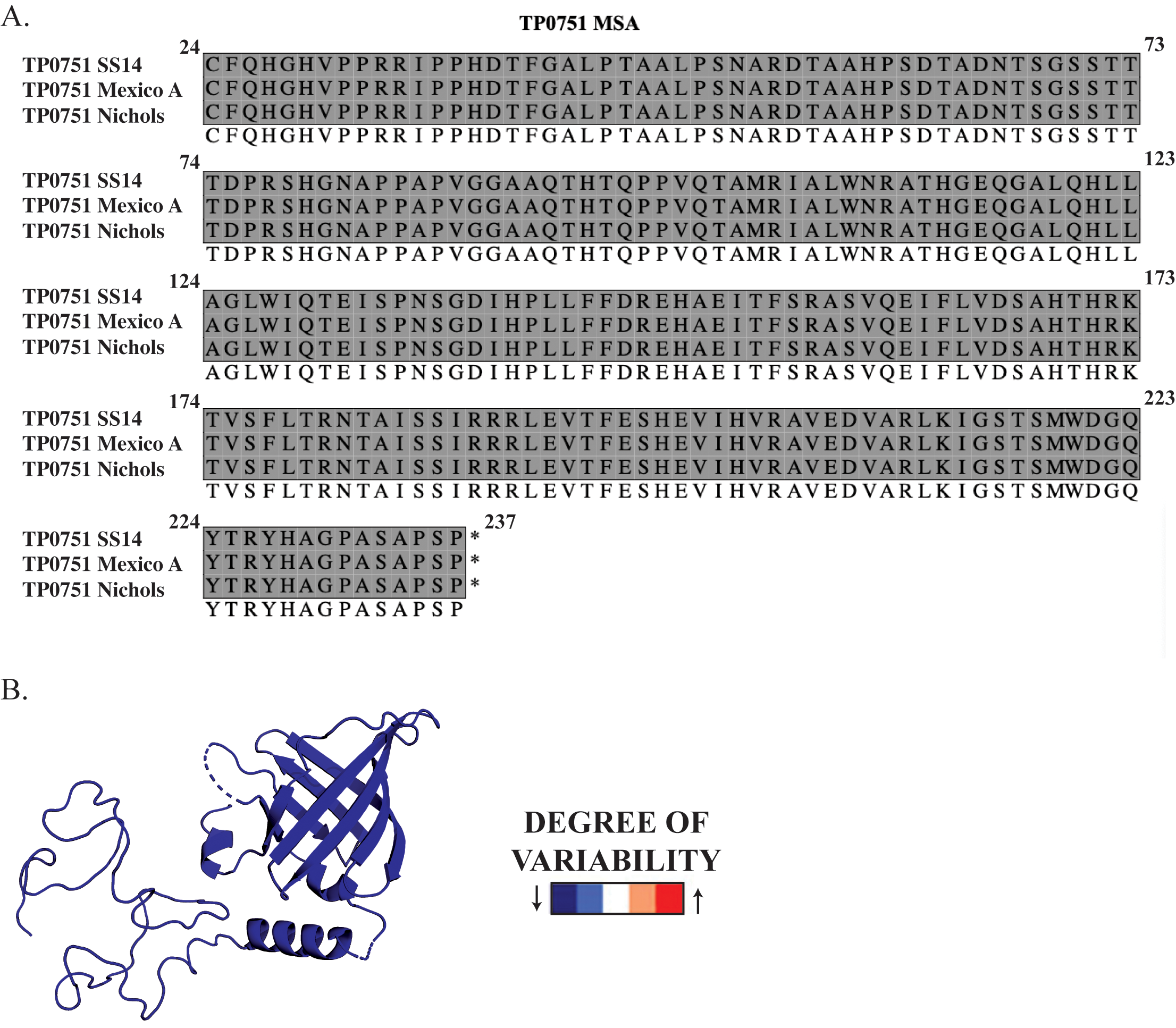

Supplement: S7 Fig — (A) Multiple sequence alignment of TP075125-237 in the Nichols, Mexico A and SS14 strains. (B) Conserved residues of TP075125-237, defined by sequence alignments of 32 different strains of T. pallidum, superimposed on the homology model and colored according to Shannon entropy scores. (TIF) [file ppat.1008871.s007.tif]

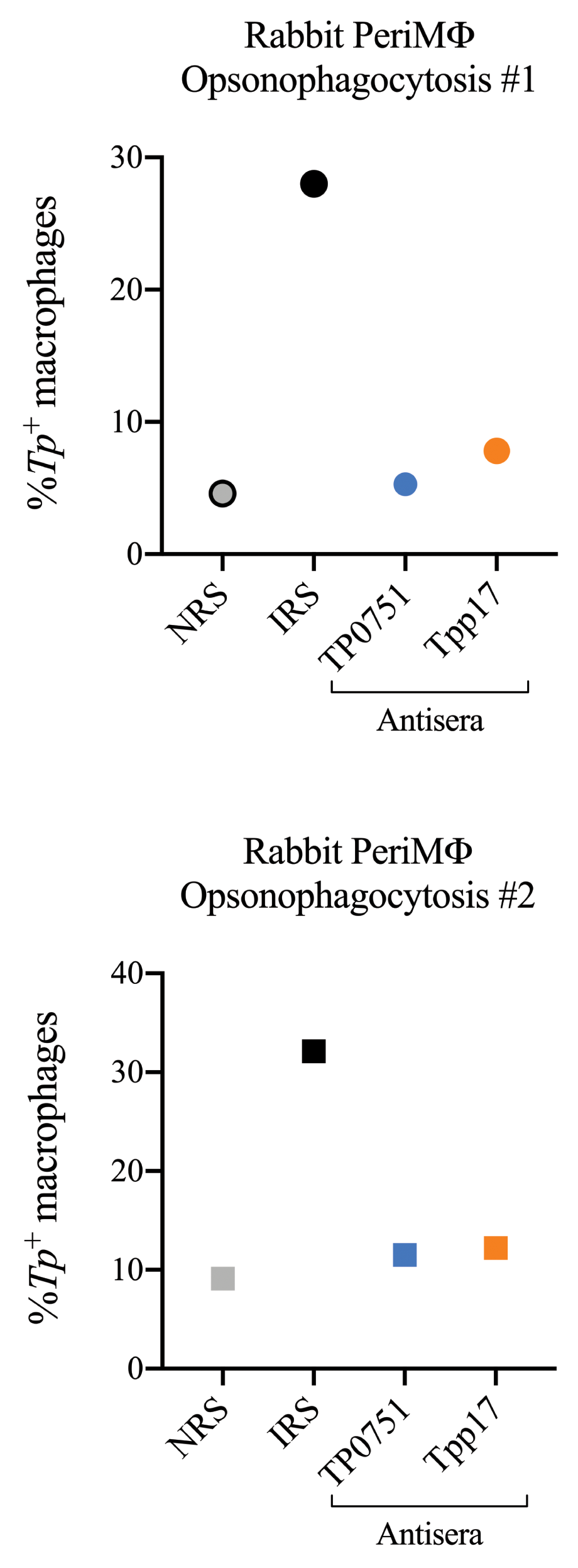

Supplement: S8 Fig — Freshly extracted T. pallidum were incubated with 10% heat-inactivated normal rabbit serum (NRS), immune rabbit serum (IRS), or rabbit antisera to TP075125-237 or Tpp17 for 2 h prior to incubation with rabbit peritoneal macrophages (PeriMΦ) for 4 h at an MOI 10:1. Following incubation, T. pallidum and nuclei were labeled as described in Section “Materials and Methods.” Spirochetal uptake was quantified as % T. pallidum+ (Tp) macrophages. The experiments were graphed individually. (TIF) [file ppat.1008871.s008.tif]

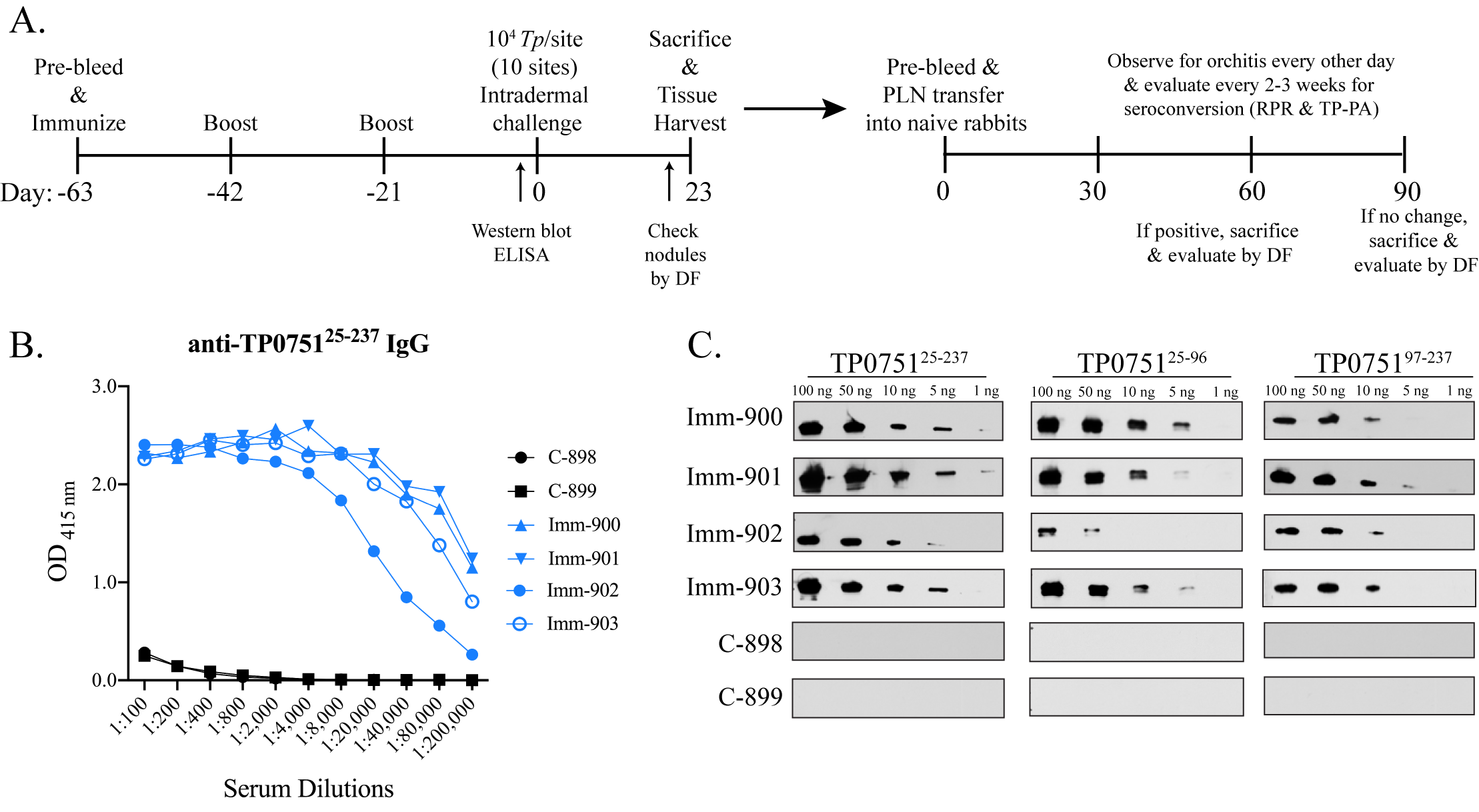

Supplement: S9 Fig — (A) TP075125-237- (n = 4) and sham-immunized (n = 2) rabbits were given four 0.1 ml subcutaneous injections into the shoulders and two 0.04 ml intramuscular injections of TP075125-237 or PBS into the quadricep muscles containing recombinant TP0751 and buffer, respectively as described by Lithgow et al. [47]. Following immunization, animals were challenged by intradermal inoculation with 1 x 10^4^ freshly extracted T. pallidum at each of 10 sites on their shaved backs. On day 23 post-challenge, animals were euthanized, and organs were harvested for qPCR assessment of bacterial burden. Popliteal lymph nodes (PLNs) were injected into the testes of naïve animals and followed for 90 days. (B) Serum antibody titers determined by ELISA (100 ng of TP075125-237 per well). (C) Reactivity of sera from immunized and control rabbits determined by immunoblot analysis against graded concentrations of TP075125-237, TP075125-96 and TP075197-237. (TIF) [file ppat.1008871.s009.tif]

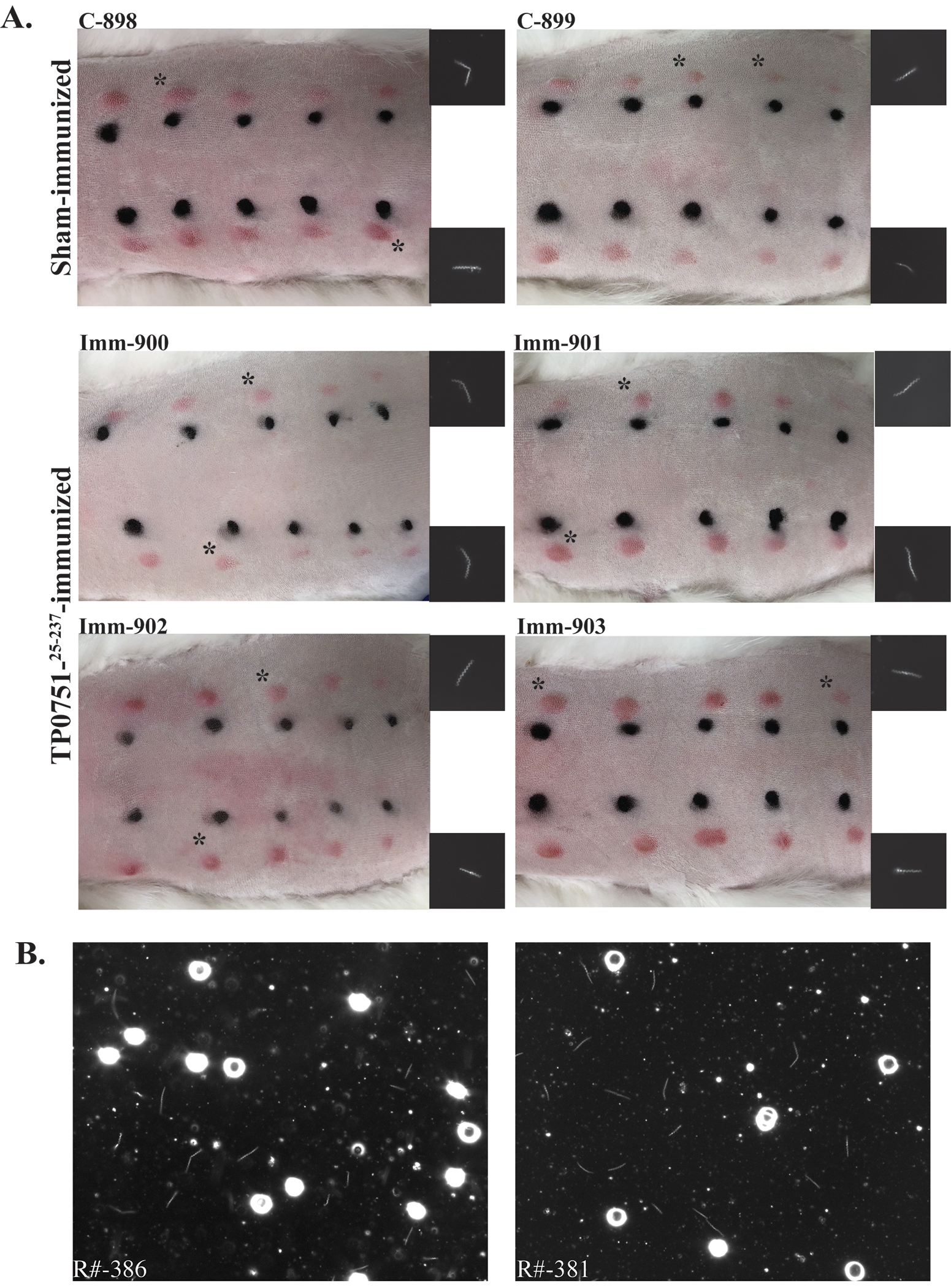

Supplement: S10 Fig — (A) Cutaneous lesions for TP075125-237- and sham-immunized rabbits on day 17 post-challenge. Aspirates were collected from two sites (indicated asterisks) and assessed by DF microscopy (insets on right). (B) Representative DF micrographs (60X magnification) of the testicular exudate from rabbit #386 (sham-immunized) and #381 (TP075125-237-immunized PLN recipient) on day 45 post-PLN transfer. (TIF) [file ppat.1008871.s010.tif]
